# Bridging the gap: Implementing BRIDGES (Brussels Resilience and Inclusion for Depression - Guidance, Exercise and Support) to shape adolescent outpatient mental health care. A pilot randomized controlled study protocol

**DOI:** 10.1371/journal.pone.0360292

**Published:** 2026-10-27

**Authors:** Arnaud Philippot, Irène Ibanez, Marine Coppens, Coralie Eeckhout, Catherine Cescutti, Alexandre Beine, Esther Meers, Tine Van Damme, Marc Francaux, Vincent Dubois, Davy Vancampfort

**Affiliations:** 1 Department of Rehabilitation Sciences, KU Leuven, Leuven, Belgium; 2 AREA+, Psychiatric Units for Adolescents, Epsylon ASBL Psychiatric Network, Brussels, Belgium; 3 UCLouvain, Louvain-la-Neuve, Institute of Neuroscience, Belgium; 4 University Psychiatric Center KU Leuven, Leuven, Belgium; 5 UCLouvain, Bruxelles-Woluwe, Institute Health and Society, Belgium; 6 Leuven Brain Institute, Leuven, Belgium; PLOS: Public Library of Science, UNITED STATES OF AMERICA

## Abstract

**Introduction:**

Physical activity interventions for adolescents with depression often show limited effectiveness due to insufficient developmental tailoring, limited autonomy support, and poor contextual feasibility. BRIDGES (Brussels Resilience and Inclusion for Depression – Guidance, Exercise, and Support) is a theory-informed and co-designed physical activity intervention integrating the COM-B model, self-determination theory and motivational interviewing to support autonomous and competence-enhancing engagement. This pilot randomized controlled trial uses a hybrid type 2 implementation–effectiveness design to examine feasibility, acceptability, implementation processes and obtain preliminary estimates of clinical effectiveness to inform a future larger-scale trial.

**Methods and analysis:**

Sixty physically inactive adolescents aged 12–19 years with depression will be recruited from an outpatient mental health service in Brussels, Belgium. Participants will be randomized in waves using covariate-adaptive minimization with a biased-coin component (p=0.85), balancing baseline depression severity, physical activity level, gender and age. The intervention consists of 18 individualized physiotherapist-led counselling sessions delivered over 12 weeks, including structured support to facilitate transition to community-based physical activity. Primary feasibility, acceptability, implementation outcomes will be assessed using quantitative measures and semi-structured interviews with adolescents, caregivers and community partners, guided by the Practical, Robust Implementation and Sustainability Model (PRISM) and the Reach, Effectiveness, Adoption, Implementation and Maintenance (RE-AIM) framework. Preliminary effectiveness outcomes are changes in physical activity measured using the Physical Activity Vital Sign (PAVS) and depressive symptoms measured using the Patient Health Questionnaire-9 modified for Adolescents (PHQ-A). Preliminary effectiveness will be analysed using linear mixed-effects models following an intention-to-treat approach to estimate cluster-level parameters for a future fully powered Hybrid Type 2 trial. Secondary outcomes include social support, exercise self-efficacy, cardiorespiratory fitness and waist-to-height ratio. Qualitative data will be analysed using the Qualitative Analysis Guide of Leuven.

**Trial registration:**

ClinicalTrials.gov NCT07422909

## Introduction

Engagement in physical activity has well-documented physical and mental health benefits for adolescents with depressive disorders [1–3], particularly in highly structured inpatient settings, where participation is supervised and embedded in routine care, leading to measurable improvements in depressive symptoms and cardiorespiratory fitness [4]. However, these benefits rarely translate into sustained physical activity once adolescents transition to less structured outpatient or community-based mental health care [5]. Existing interventions for outpatient adolescents often lack a strong theoretical foundation and are insufficiently tailored to developmental stage, individual preferences, or real-world context. Critical gaps include inadequate support for adolescents’ autonomy (opportunities to make choices about activity), motivation (fostering intrinsic engagement rather than relying on external pressure), and environmental support (availability of safe, accessible spaces and social encouragement for activity) [6–8]. Consequently, interventions frequently demonstrate limited effectiveness, low uptake, and substantial implementation challenges, contributing to persistently low physical activity levels after discharge and during outpatient care [5,9]. These low activity levels increase cardiometabolic risk in an already vulnerable population [10,11] and further compound long-term physical and mental health burdens into adulthood [11–13].

To address these gaps, we co-developed a multi-component, theory-informed physical activity intervention specifically for outpatient adolescents with depression, named BRIDGES for *In Brussels, Resilience and Inclusion for Depression trough Guidance, Exercise and Support* [8]. The development process followed the eight-step Behaviour Change Wheel framework (BCW), underpinned by the Capability, Opportunity, Motivation – Behaviour (COM-B) model [14], and incorporated participatory co-design with adolescents [15,16]. According to COM-B, adolescents require capability (psychological and physical capacity), opportunity (social and environmental enablers), and motivation (reflective and automatic processes) to enact behaviour change. These constructs provide a structured framework for identifying and targeting the multidimensional determinants of physical activity engagement in adolescents with depression.

Social connectedness, adequate support, and shared decision-making emerged as central drivers of engagement during the co-design process [8]. In response, the intervention incorporates structured social opportunities and applies evidence-based principles from self-determination theory [17] and motivational interviewing [18] to address autonomy, competence, and relatedness. Through these mechanisms, the intervention fosters intrinsic motivation and creates autonomy-supportive environments conducive to sustained physical activity engagement [19,20]. The multi-component program combines physiotherapist-led counselling with structured support to transition adolescents into community-based activity [21].

Given that poor uptake of physical activity interventions has been identified as a key barrier in Belgian outpatient psychiatric care (KCE 338, p.176, § 8.2.3.3), the intervention was deliberately designed to align with the Belgian reimbursement structure (up to 18 physiotherapy sessions of 30 minutes each per year), supporting feasibility, scalability, and integration into routine care. By explicitly linking COM-B constructs to intervention components, the model clarifies hypothesized causal pathways through which behaviour change is expected, enhancing transparency, fidelity, and replicability [14,17,18].

The BRIDGES intervention as a co-designed care pathway is newly developed [8] and its core clinical component, physical activity as a treatment for adolescent depression, is supported by a substantial and growing body of evidence demonstrating beneficial effects on depressive symptoms and physical health [2–5,22,23]. In parallel, the Lancet Psychiatry Physical Health Commission has emphasized shifting research focus from efficacy alone to implementation, highlighting the need to understand how lifestyle interventions can be integrated into routine mental health care [11]. In line with this agenda, a pilot hybrid type 2 implementation–effectiveness study is warranted, enabling evaluation of preliminary effects on physical activity and depressive symptoms, as well as feasibility, acceptability, fidelity, and contextual factors influencing implementation in outpatient services [11].

Beyond effects on physical activity levels and symptoms, the intervention may facilitate new social connections within adolescents’ communities [24] and enhance exercise self-efficacy through physiotherapist-led counselling. Social support and self-efficacy are central determinants of health behaviour adoption [14], underpin autonomous motivation [17], and are associated with improved mental health outcomes [21], justifying their inclusion as secondary outcomes.

Low cardiorespiratory fitness is a major cardiometabolic risk factor in adolescents with depressive symptoms [25]. Incorporating objective assessments of changes in physical fitness over time strengthens the pragmatic evaluation of intervention impact, ensuring relevance to real-world outpatient care beyond standardized efficacy measures.

The primary aim of this pilot study will evaluate implementation strategy outcomes, including, barriers and facilitators to delivery, and potential for long-term integration within existing care pathways, fidelity to motivational interviewing and self-determination theory principles and coordination between outpatient services and community-based resources. Simultaneously, the study will be to assess the preliminary effectiveness of the BRIDGES intervention on physical activity levels and depressive symptoms among outpatient adolescents with depression, compared with a waitlist control receiving usual care. The pilot data will be used to estimate cluster-level parameters and calculate the required number of implementation sites for a future fully powered Hybrid Type 2 trial. Secondary outcomes will include perceived social support, exercise self-efficacy and cardiorespiratory fitness providing a comprehensive understanding of mechanisms underlying behaviour change and mental and physical health improvement.

## Methods

### Trial registration

This trial has been registered at ClinicalTrials.gov (SI 1) under registration number: NCT07422909. The registration includes the study design, intervention details, outcome measures, and planned analyses, in accordance with the SPIRIT 25 guidelines [26] to ensure transparent, complete, and reproducible reporting of the planned trial procedures (SI 2). The study will begin in March 2026 and will finish in October 2027.

### Design and participants

This study will use a pilot hybrid type 2 implementation–effectiveness randomized controlled trial design, allowing simultaneous the processes supporting BRIDGES intervention uptake in routine outpatient care evaluation of its impact on adolescents [27]. The BRIDGES intervention comprises 18 individualized sessions delivered over 12 weeks, combining physiotherapist-led counselling with structured support to transition into community-based physical activity. Firstly, implementation measures will be guided by the Practical, Robust Implementation and Sustainability Model (PRISM) [28] and the Reach, Effectiveness, Adoption, Implementation, and Maintenance (RE-AIM) framework [29], which together provide a structured approach to assess how the intervention is delivered, adopted, and sustained across outpatient and community-based settings. Secondly, primary outcomes are physical activity and depressive symptoms; secondary outcomes include perceived social support, exercise self-efficacy, and cardiorespiratory fitness. This dual perspective enables a comprehensive understanding of both the intervention’s effects and the factors influencing its successful integration into real-world clinical practice. Participants will be inactive adolescents aged 12–19 years with a confirmed diagnosis of depression, receiving outpatient care at AREA + , a specialized adolescent mental health network in Brussels. Adolescents from AREA+ experience a range of psychological difficulties, including depression, anxiety, school dropout, family crises, psychosocial vulnerabilities, risky or suicidal behaviours, and psychotic symptoms. Eligibility and exclusion criteria are defined according to the PICO framework (Table 1). Key inclusion criteria include voluntary participation, depression diagnosis, ability to stay focused for 30 minutes, fluency in French, physical inactivity as defined by WHO guidelines [30], and residence in Brussels. Exclusion criteria include intellectual disability (IQ < 70) and serious medical conditions or precautions that contraindicate higher-intensity exercise, as determined by the Physical Activity Readiness Questionnaire (PAR-Q) and, if evaluated necessary by the PAR-Q, confirmed by a general practitioner.

**Table 1:** PICO-criteria.

PICO	Criteria
**P**articipants	All adolescents aged 12–19 with a diagnosis of depression who are receiving outpatient care through AREA + . Other inclusion criteria are: (a) voluntary participation and accepting the randomisation, (b) being admitted at AREA + , (c) being able to stay focused for 30 minutes, (d) having a good knowledge of French, (e) physically inactive defined as not meeting the PA guidelines from World Health Organisation [74] following the corresponding age (i.e., at least 420 min of moderate to vigorous physical activity (MVPA) /week for those aged 12–17 and at least 150 min of MVPA /week for those aged 18 and 19 years, (f) living in Brussels. Exclusion criteria are (a) intellectual disability (defined as an intelligence quotient < 70); and (b) participants with serious medical problems or medical precautions prohibiting higher-intensity exercise (e.g., severe metabolic disorders, oncologic disease, unstable diabetes) according to the recommendations of the American College of Sports Medicine. Physical Activity Readiness Questionnaire – PAR-Q will be used for adolescents for checking conditions and precautions from age of 15. For teenagers under 15 and if an older adolescent obtains a “yes” to the PAR-Q, a general practitioner will review the medical conditions and precautions to this end.
**I**ntervention	Physical activity counseling by a physiotherapist during 18 individual 30 min sessions, based on motivational interviewing and self-determination principles.
**C**ontrol condition	Wait list. Outpatient care as usual.
**O**utcomes	The main outcomes are changes in physical activity levels measured with the Physical Activity Vital Sign (PAVS), symptoms of depression as measured with the Patient Health Questionnaire – 9 – adolescents version (PHQ-A) in the experimental group vs control group every month. Secondary outcomes will include assessments of four dimensions of depression (depressive mood, low self-evaluation, somatic complaints, social withdrawal), resilience, social support, self-efficacy, and cardiorespiratory fitness at baseline and three months post-intervention. These will be measured using Beck Depression Inventory II (BDI II), Multidimensional Scale of Perceived Social Support (MSPSS), the adapted Self-Efficacy for Exercise Scale (SEE)^1^, and the Åstrand-Ryhming Test (ART).

The overall study flow is summarized in Fig 1 with the SPIRIT [31] timeline, including recruitment, participant, intervention, and assessment.

**Fig 1:**
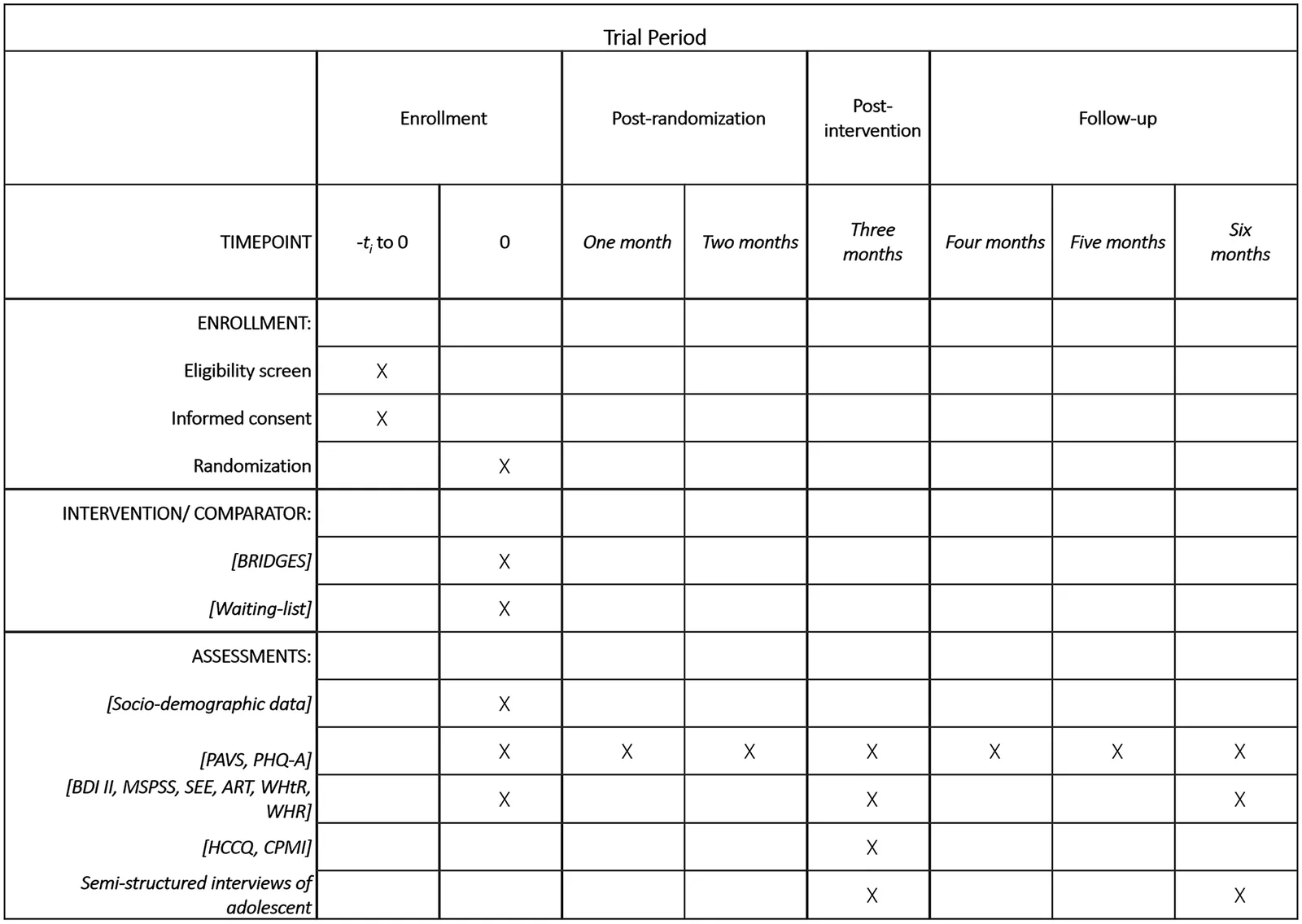
Participant timeline: Schedule of patients enrollment, interventions, and assessments. Legend: PAVS = Physical Activity Vital Sign; PHQ-A = Patient Health Questionnaire-9 modified; BDI-II = Beck; Depression Index; MSPSS: Multidimensional Scale of Perceived Social Support; SEE: adapted Self-Efficacy for Exercise Scale; ART = Astrand Rhyming test; WHtR = Waist-to-height ratio; WHR = Waist-to-hip ratio; CPMI = Client Perception of Motivational Interviewing; HCCQ = Health Care Climate Questionnaire.

### Recruitment procedure

The study will be conducted at the AREA+ outpatient service, Consult’ados, which provides follow-up care for adolescents after AREA+ hospitalization, as well as for new external referrals seeking outpatient treatment. Adolescents meeting the inclusion criteria and expressing interest will be scheduled for a consultation with the physiotherapist delivering the BRIDGES intervention. Participants will be encouraged to bring a trusted caregiver or supportive person to support informed decision-making. For minors, parents or legal guardians will be actively involved in the consent and assent process. Recruitment will occur in waves over nine months through December 2026, with the intervention delivered over 12 months through March 2027 and follow-up data collection and completion continuing for 15 months through June 2027, including a three-month post-intervention assessment. The results are expected in July 2027.

### Ethical considerations

The study will be conducted in accordance with ethical principles outlined in the Declaration of Helsinki [32] and applicable national regulations [33,34]. All participants, along with their parents or legal guardians for minors, will receive comprehensive, age-appropriate information about the study’s objectives, design, procedures, potential risks and benefits, and data management practices. Participant privacy will be strictly protected through pseudonymization, with identifiable data accessible only to the research team and authorized representatives. Informed written assent/consent will be obtained from all participants prior to enrolment. For adolescents under 18 years, written consent will also be obtained from a parent or legal guardian. The assent/consent process will include a dedicated session explaining the study step-by-step, allowing ample time for questions and discussion to ensure fully informed voluntary participation. Participants and their guardians will be explicitly informed of their right to withdraw at any time without any consequences to their care. Adverse events, including injuries or symptom exacerbation during sessions, will be monitored and recorded at each session. Serious adverse events will be reported to the ethics committees within 24 hours. Then, if the participant, as well as a parent or legal guardian in the case of minors, agree to participate, they must complete and sign a written consent form. For individuals who do not provide consent or assent to participate in the intervention, but who previously provided written consent at admission for their clinical data to be used for research purposes, demographic and relevant clinical data will be used in aggregate to assess the representativeness of the study population and to compare characteristics of participants versus non-participants. The study protocol has been reviewed and approved by the Saint-Luc University Hospital Ethics Committee – UCL (B4032024000098) and the local ethics committee of Epsylon, including AREA + , ensuring compliance with national and institutional ethical standards (SI 3). All study procedures, including recruitment, consent, data collection, and data storage, will adhere to these approved protocols. Any important modifications to the study protocol (e.g., changes in eligibility criteria, procedures, or data collection methods) will be communicated promptly to the ethics committee, and the study sponsor. Protocol amendments will also be updated in the trial registry as required (ClinicalTrials.gov).

### Randomisation process

A total of 60 participants will be recruited in small waves. Enrollment will be conducted by the research coordinator following confirmation of eligibility and completion of baseline assessments. To ensure balance across key baseline characteristics between the BRIDGES intervention and waitlist control (treatment-as-usual, TAU) groups, a covariate-adaptive minimization method with a random component (biased-coin probability, p = 0.85) will be employed. Minimization is a dynamic, adaptive randomization technique in which each new participant is assigned in real time based on their baseline characteristics, rather than using a predetermined sequence. This method optimizes balance across important covariates while preserving unpredictability of assignment. Covariates included in the minimization algorithm are depression severity (Patient Health Questionnaire-9- modified for adolescent, PHQ-A), physical activity level (Physical Activity Vital Sign, PAVS), gender (male, female, other), and age group (12–15 years or 16–19 years). Continuous variables will be standardized to reduce the influence of extreme values, with greater weight given to depression severity and physical activity, while still accounting for gender and age. The allocation sequence will be generated automatically using R software (R 4.5.2), functioning as a secure “black box”, stored by an external researcher. Upon enrollment, the automated system will assign each participant to either the BRIDGES intervention or the waitlist control, designated as Group A or Group B. The external researcher will remain blinded to participant identity and group assignment. Investigators conducting baseline assessments and delivering the intervention will be informed of the labels but will remain blinded to the overall allocation, ensuring allocation concealment and minimizing selection bias. Participants assigned to the control group will receive €20 vouchers at baseline, post-intervention, and three-month follow-up, while retaining the option to participate in the intervention outside the study. Participants in the experimental group will receive a €20 voucher at follow-up. This modest incentive aims to enhance retention and adherence while maintaining ethical transparency.

### Intervention

The BRIDGES intervention offers a comprehensive biopsychosocial-spiritual approach to support adolescents with depression in integrating physical activity into their daily lives (Fig 2). It was developed in direct response to adolescent input, emphasizing a true sense of choice and personalization in how the program is delivered. The initial sessions introduce the intervention, foster open discussion, and create a supportive environment for learning and exchange between the adolescent and the physiotherapist-counsellor. At the outset, session frequency and delivery mode (online or face-to-face) will be determined according to each adolescent’s preferences over the three-month program, allowing up to two remote sessions for every face-to-face session, in line with the Belgian physiotherapy framework [35]. The program combines individualized counselling with a structured transition to community-based physical activity opportunities. Counselling sessions, led by trained physiotherapists, are grounded in evidence-based behaviour change frameworks, with particular emphasis on motivational interviewing and need-supportive coaching grounded in the self-determination theory [17]. These approaches are particularly suitable for adolescents with depression, as they enhance internal motivation, autonomy, and personal agency. Motivational interviewing techniques facilitate collaborative, non-judgmental dialogue, helping adolescents resolve ambivalence about physical activity. Through reflective listening and open-ended questions, physiotherapists guide participants to explore their values, goals, and motivations, fostering a sense of ownership over the behaviour change process. Drawing on the self-determination theory, counselling targets three key psychological needs: autonomy (control over one’s choices), competence (feeling capable and effective), and relatedness (feeling connected and supported). Sessions are tailored to each adolescent’s readiness, preferences, and personal circumstances, addressing individual barriers and strengths. Relevant opportunities from the AREA+ network, including local sports clubs and initiatives, are introduced in ways that align with adolescents’ interests and intrinsic motivation. To support transition into community-based physical activity, a network of inclusive clubs and associations has been mapped in the AREA+ region. Adolescents are guided toward independent participation through a flexible, stepwise process, which may include meeting coaches, exploring settings, trial periods, and trying out different clubs within the 18-session program. Throughout this process, ongoing counselling provides consistent guidance, motivation, and emotional support. All participating clubs and associations are carefully selected and provided with targeted training, requested during the co-design phase, to ensure they can effectively support adolescents with depression. Training focuses on trauma-informed and psychologically safe physical activity practices [36–38] to foster inclusive and accessible participation. The implementation and effectiveness of this training are being evaluated in a separate study.

**Fig 2:**
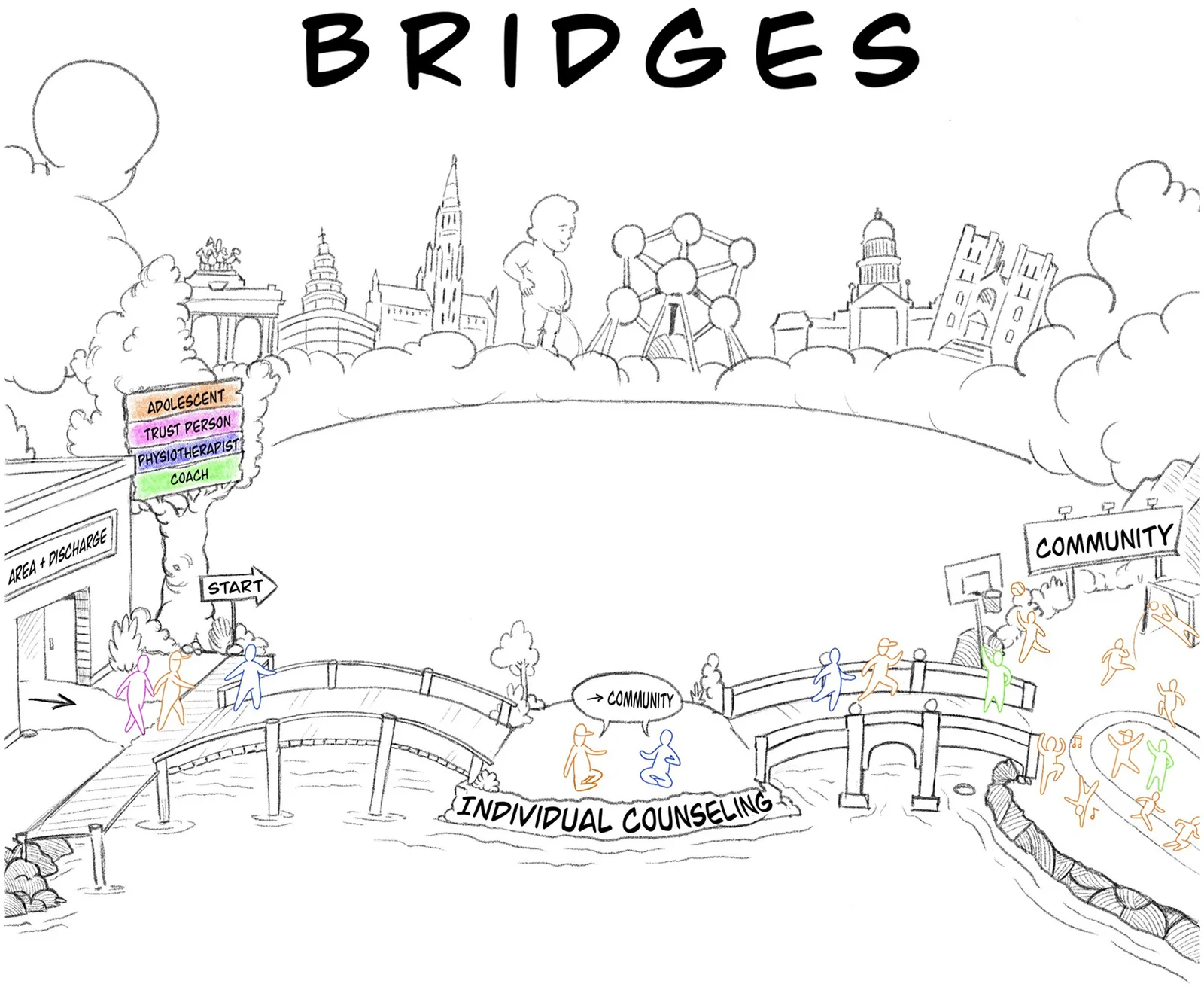
Modified illustration of BRIDGES (Brussels Resilience and Inclusion for Depression – Guidance, Exercise and Support) intervention, co-designed with inpatient adolescents [8] (illustrator, Carl Vervier).

Accordingly, the comparator group will be a waitlist control receiving usual care, allowing evaluation of the added benefit of the intervention within the existing care context. The usual standard outpatient care, which may include psychotherapy, medication management, or other usual clinical services. No structured physical activity intervention is provided.

### Implementation measures

For the implementation assessment, PRISM will provide the overarching conceptual structure to examine organizational, individual, and system-level factors that influence delivery, uptake, and sustainability of the intervention [28]. Within this framework, the RE-AIM dimensions will be systematically applied [29]. Reach will assess the proportion and representativeness of adolescents included in the study, as well as reasons for refusal or dropout when voluntarily disclosed and exploring the barriers and facilitators to reach the intervention through staff interviews. Effectiveness will evaluate changes in physical activity, depressive symptoms, and secondary outcomes. Adoption will explore barriers and facilitators to participation and delivery from the perspectives of adolescents, clinicians, and community partners. Implementation will monitor fidelity to motivational interviewing and self-determination theory principles, a logbook with their adolescent participation and identify any adaptations made in practice. Maintenance will evaluate the potential for long-term integration of the intervention into routine care and the sustainability of the transition to community-based physical activity opportunities (Table 2). Semi-structured interviews guided by PRISM domains will complement quantitative measures, capturing perspectives of participants, clinicians, and community stakeholders (Table 3).

**Table 2:** Evaluation of the implementation using RE-AIM framework.

Concept	Measure
**R**each	Reach will be defined as the proportion and representativeness of physically inactive adolescents receiving outpatient care who consent to participate in the intervention during the specified recruitment period, including referrals from inpatient units and routine outpatient follow-up requests. The proportion will be calculated as the number of adolescents enrolled relative to the total number invited to participate. We will document the total number of outpatients screened for eligibility and record reasons for ineligibility. To assess representativeness, basic sociodemographic data will be collected for both adolescents who consent and those who decline participation, along with the stated reasons for refusal. Sociodemographic characteristics will include age, sex, gender, receipt of social financial aid, and family structure/living arrangements (two-parent household, single-parent household, or residential center). These data will be obtained from participants’ medical records, with prior assent and consent for research use provided through the AREA+ setting. In addition, semi-structured interviews with staff will be conducted to explore their perspectives on factors influencing reach, including potential barriers to recruitment and participation. This combination of quantitative and qualitative data will provide a comprehensive understanding of both the proportion and representativeness of adolescents engaged in the BRIDGES intervention.
**E**ffectiveness	Effectiveness will be evaluated both quantitatively and qualitatively. Primary outcomes include changes in physical activity levels and depressive symptoms, assessed using the PAVS and PHQ-A, respectively as described previously. Secondary outcomes will include perceived changes in physical, mental, social well-being, usual care follow-up and potential discharge which will be explored qualitatively through brief semi-structured interviews conducted post-intervention and after follow-up. Adverse events and unintended consequences will also be documented to assess intervention safety.
**A**doption	Adoption will be assessed through qualitative interviews with patients after their participation and staff from the sport clubs and healthcare staff at the end of implementation phase (12 months). These interviews will explore perceived barriers and facilitators to participating in or delivering the physical activity counselling. Insights gained will help determine the acceptability and feasibility of the intervention from multiple stakeholder perspectives, and provide a better understanding of factors influencing uptake across individuals and clinical contexts.
**I**mplementation	Implementation will focus on fidelity to the intervention protocol. Specifically, we will monitor whether key components are delivered consistently, including: [1] initial physical activity assessment during the first consultation [2], completion of the planned counselling sessions [3] ongoing participation in community-level at the follow-up and [4] lived experience of adolescents on the physical activity counseling sessions. For the second component, physiotherapists will complete a logbook documenting participants’ attendance, contextual factors, mechanisms influencing participation, and clinical observations related to adolescents’ engagement in physical activity. For this fourth part, we will be triangulated qualitative feedback from both adolescents and physiotherapists to identify contextual influences on delivery, drift, and opportunities for reinforcement during training and supervision. Deviations from the protocol, reasons for missed sessions, and adaptations made in clinical practice will also be recorded. This process evaluation will inform the degree to which the intervention was delivered as intended.
**M**aintenance	Maintenance will focus on the potential for long-term integration of the physical activity counselling program into routine clinical care. This will include assessing after 6 months whether program components are used beyond the study period, staff willingness and capacity to sustain delivery, and the extent to which the intervention aligns with existing workflows and institutional priorities. Follow-up interviews with staff and review of service delivery data (e.g., recorded physiotherapy sessions for outpatients with depression in the medical files) will support this assessment.

CPMI = Client Perception of Motivational Interviewing; HCCQ = Health Care Climate Questionnaire

PAVS = Physical Activity Vital Sign; PHQ-A = Patient Health Questionnaire-9 modified; MI = Motivational Interview; SDT = Self-Determination Theory

**Table 3:** Practical, Robust Implementation and Sustainability Model (PRISM) domains addressed in the semi-structured interviews.

PRISM domain	Participant groups	Examples of questions
Implementation structure	physiotherapist	What ideas do you have for maximizing efficiency in the workflow for (a) the assessments, (b) the brief physical activity counseling intervention?
External environment	Clinicians and management	I’m interested in learning about your impressions of the current climate about physical activity counseling as part of a holistic care for adolescents outpatients here. In your opinion, do you believe your setting has the capacity for incorporating an initiative like the one we executed in this pilot study? Considering the climate as well as the potential benefits and barriers of this initiative, what are your thoughts regarding the implementation of the intervention?
Clubs/assocations in community-level	Coachs /physical activity provider	I’m interested in learning about your impressions of the inclusion of adolescents with depression that you welcomed. In your opinion, do you believe your setting and your personnel has the capacity for incorporating an initiative like the one we executed in this pilot study?
Intervention design	Patients	What do you like or dislike about the current intervention? Could you give feedback or suggestions about potential adaptations you consider of added value? How do these sessions support your motivation?

### Fidelity

#### Motivational interviewing fidelity.

Fidelity to motivational interviewing (MI) will be assessed using a combination of observer- and participant-reported measures. First, adherence to MI principles will be evaluated using the Independent Tape Rater Scale (ITRS), which is recommended for effectiveness studies [39] (Hurlocker et al., 2020). The ITRS assesses the extent to which practitioners apply MI in real-world, community-based settings [40]. The scale comprises 39 items evaluating the use of MI-consistent techniques (e.g., open-ended questions, affirmations) and MI-inconsistent strategies (e.g., direct confrontation), as well as general counselling skills. Of these, 10 items specifically assess MI-consistent behaviours and are further categorized into basic skills (e.g., reflective listening) and advanced skills (e.g., eliciting and strengthening discrepancy). Each item is rated on a seven-point Likert scale across two dimensions: adherence (1 = not at all to 7 = extensively) and competence (1 = very poor to 7 = excellent). A randomly selected sample of physiotherapist-led counselling sessions, recorded with adolescent consent, will be transcribed and independently coded by trained raters. Both global ratings (e.g., empathy, collaboration) and behaviour counts (e.g., open-ended questions, reflections, affirmations) will be derived. To complement observer ratings, adolescents will complete the Client Perception of Motivational Interviewing (CPMI) scale [41], which captures participants’ experiences of MI-consistent communication during sessions. This combined approach enables assessment of both the technical and relational components of MI fidelity while remaining feasible within the constraints of a pilot implementation study.

#### Self-Determination theory fidelity.

Fidelity to self-determination theory will be assessed using participant-reported measures aligned with its core constructs: autonomy, competence, and relatedness. Adolescents will complete the Health Care Climate Questionnaire (HCCQ) [42] to evaluate perceived autonomy support provided by the physiotherapist. In addition, brief post-session feedback forms will invite participants to rate the extent to which they felt understood, empowered, and supported in setting their own goals—key indicators of SDT-congruent delivery.

Fidelity data will be triangulated with qualitative feedback from both adolescents and physiotherapists in PRISM evaluation to identify contextual influences on intervention delivery, potential drift over time, and opportunities for reinforcement through training and supervision. Maintaining high fidelity to MI and SDT principles will be considered a core implementation outcome, given its direct relevance to the hypothesized mechanisms of action underlying behaviour change.

### Effectiveness outcomes and measures

Data collection will occur after confirmation of eligibility and participant consent. The first visit, conducted prior to randomization, will assess baseline outcomes (T0) and collect socio-demographic information. Subsequent visits will occur post-intervention (T3) and at three-month follow-up (T6). Monthly assessments of physical activity (PAVS) and depressive symptoms (PHQ-A) will be completed remotely at T2, T3, T4, and T5 (Fig 1). All data will be entered directly through a web browser into a secure REDCap server, hosted by the university and maintained according to high-level security and backup standards.

### Socio-demographic characteristics

We will collect socio-demographic and clinical data from participants, including age (years), gender (female, male, other), current medications (name and, if available, dosage in mg), duration of school dropout (months), and family structure (two parents, single parent, or under guardian care). Clinical information will include duration of current depressive episode (months), lifetime history of depression (number of previous episodes), socio-economic status with the Family Affluence Scale III [43], co-occurring mental health conditions (e.g., anxiety, ADHD, psychosis, PTSD), and relevant somatic or physical health conditions (e.g., diabetes, asthma).

### Primary and secondary outcomes

Primary and secondary outcomes have been selected to capture both the effectiveness of the BRIDGES intervention on adolescents’ physical activity and mental health, as well as its broader psychosocial and physiological effects. Primary outcomes, focusing on physical activity behavior and depressive symptoms, will be assessed at baseline, every 4 weeks during the intervention and again three months post-intervention to monitor changes over time. Secondary outcomes will be measured at baseline, post-intervention, and three-month follow-up.

### Primary preliminary effectiveness measures

#### Physical Activity Vital Sign (PAVS).

The PAVS is a screening tool used to assess patients’ physical activity levels [44]. It consists of two questions: (a) on average, how many days per week do you engage in moderate to vigorous physical activity, and (b) on those days, how many minutes do you typically spend participating in moderate to vigorous physical activity? The product of these responses (days × minutes) provides an estimate of the patient’s average weekly minutes of physical activity in moderate to vigorous physical activity. This screening tool is also sensitive to changes in mental health care trial [45] and able to identify physical inactivity associated with cardio-metabolic risk factors in youth with mental illness [46,47].

#### Patient Health Questionnaire-9 modified for Adolescents (PHQ-A).

The PHQ-A is a brief instrument modified to adolescents used to assess the severity of depression [48]. It contains 9 items that are on a scale from 0 to 3, with the total score ranging from 0 to 27. Adapted from the Diagnostic and Statistical Manual of Mental Disorders, Fourth Edition (DSM-IV), the PHQ-A encompasses the same diagnostic symptom criteria outlined in the DSM-IV, including, the two cardinal signs of depression (anhedonia and depressed mood), cognitive symptoms and physical symptoms [49]. The PHQ-A is shorter than many other depression screening tools and can be self-administered. It’s a valid and reliable assessment tool for the severity of depressive symptoms in adolescents with major depression disorder [48] and is sensitive to changes among adolescents in psychiatric setting with reliable change index of 7 [50]. Compared to semi-structured interview reference standard, pooled PHQ-A sensitivity and specificity (95% confidence interval) at the standard cut-off value of ≥10 were 0.85 (0.79 to 0.89) and 0.85 (0.82 to 0.87) [51].

### Secondary outcomes

#### Beck Depression Index (BDI-II).

The BDI-II captures specific behavioural manifestations of depression. It is a 21-item self-report inventory, with each item scored from 0 to 3 based on four statements reflecting symptom severity, yielding a total score ranging from 0 to 63. The BDI-II has demonstrated validity and reliability in adolescents with major depression [52,53]. Internal consistency has been reported around 0.9, and test-retest reliability ranges from 0.73 to 0.96 in adolescent populations, including psychiatric settings [54,55]. Sensitivity and specificity at a cutoff score of 16 were 73.0% (95% CI: 62.0–81.8%) and 80.3% (95% CI: 72.8–86.1%), respectively [55]. While the PHQ-A is brief, time-efficient, and well-suited for frequent, repeated assessments [48] during the intervention, it primarily captures overall depressive symptom severity and is less sensitive to nuanced changes in cognitive, affective, and behavioural domains. In contrast, the BDI-II provides a more detailed and multidimensional assessment, including subtle emotional, motivational, and somatic symptoms, such as negative self-perception, guilt, and changes in sleep or appetite, that may not be fully reflected in the PHQ-A. Administered at baseline, post-intervention, and three-month follow-up, the BDI-II complements the PHQ-A by allowing a deeper evaluation of the trajectory and persistence of depressive symptoms over time, while the PHQ-A enables practical, high-frequency monitoring of short-term changes during the 12-week intervention [52]. Together, these measures balance feasibility with sensitivity, providing a comprehensive understanding of intervention effects on adolescent depression [52].

#### Multidimensional Scale of Perceived Social Support (MSPSS).

The Multidimensional Scale of Perceived Social Support (MSPSS) is a widely used self-report instrument designed to assess an individual’s perception of social support from different sources [56]. The scale comprises 12 items, each rated on a 7-point Likert scale ranging from a score of 7–84. Rather than conceptualizing social support as a single global construct, the MSPSS adopts a multidimensional approach and is structured around three distinct subscales: support from a significant other, family, and friends. The MSPSS has been extensively validated across diverse populations and age groups. Previous research has consistently reported high internal consistency for the total scale as well as for each of the three subscales among young people, including those involved in well-being and prevention programs [57,58] and has demonstrated reliability and sensitivity in outpatient and psychiatric setting [59]. These findings support the MSPSS as a reliable and robust measure for assessing perceived social support in both community and clinical youth populations.

#### Adapted Self-Efficacy for Exercise Scale (SEE).

Exercise self-efficacy (SEE) is a key determinant of intention formation and engagement in physical activity. In the original SEE, respondents rate their confidence in exercising three times per week for 20 minutes. For the present study, the scale was adapted so that the target behaviour reflects an individualized exercise goal defined by each adolescent, that is, the type of physical activity they wish to perform. This adaptation enhances developmental relevance and ecological validity while retaining the standardized structure and response format of the original items. The original SEE has demonstrated high internal consistency and validity. The SEE consists of nine items rated on an 11-point Likert scale [60] and has demonstrated excellent internal consistency and validity (Cronbach’s α = 0.92; McDonald’s ω = 0.81) [61], including adolescent populations [61].

### Physical fitness

#### Astrand Rhyming test (ART).

ART is a single-stage cycle ergometer test, to measure the cardiorespiratory fitness levels (assessed as an estimation of VO2 max) [62]. It is designed to elicit a steady-state heart rate over a 6-min period. The initial workload will be selected from the Astrand test loading wattage table, pedalling speed remains constant (60 revolutions per min) and heart rate (HR) is recorded with a Polar heart rate sensor (Polar Electro Oy, Kempele, Finland) at every minute interval. The workload is set during the first 2 min and maintained throughout the test. In the case that HR fails to achieve the target zone, the load is adjusted accordingly. HR and loading wattage are noted at the end of each minute, with a target goal of obtaining two consecutive HR values over 120 beats but below 170 beats per minute (bpm), within 5 bpm of each other, during the fifth and sixth minute of work. The protocol will be interrupted if threatening symptoms appear or when the HR reached 85% of age-predicted (220-age) maximum HR [11]. The test-retest reliability was 0.92 (95% confidence interval: 0.85–0.95) in adults with schizophrenia [63] and in adolescents with autism spectrum disorder (95% confidence interval: 0.84–0.85) [64], including moderate to strong levels of association (*r*  =  0.74–0.75) with a maximal cardiopulmonary exercise test [64].

#### Waist-to-height ratio (WHtR).

WHtR is calculated as waist circumference (cm) divided by height (cm). Waist circumference will be measured in standing position, midway between the lowest border of the rib cage and the superior border of the iliac crest, at the end of a normal expiration. Three waist circumference measurements will be taken with a non-stretchable tape and the mean value is used in the WHtR calculation. The universal WHtR cut-off of 0.5 will be used to identify participants at increased cardiometabolic risk [65]

#### Waist-to-hip ratio (WHR).

WHR is calculated as waist circumference (cm) divided by hip circumference (cm). Waist circumference is measured in a standing position, midway between the lowest border of the rib cage and the superior border of the iliac crest, at the end of a normal expiration. Three waist circumference measurements will be taken with a non-stretchable tape and the mean value is used in the WHtR calculation. It serves as a key indicator of central obesity and metabolic syndrom risk, with gender-specific thresholds > 0.89 [66]. Unlike BMI, WHR distinguishes between abdominal and peripheral fat, offering a stronger predictive value for obesity-related complications.

### Data analysis

#### Power analysis.

For the qualitative component, guided by the PRISM framework, we will recruit a purposive subsample of approximately 12 participants per stakeholder group, including adolescents, clinical staff (physiotherapists and collaborating psychologists, social workers, psychiatrists, and educators), managers, and community-based physical activity partners within the care network. This sampling strategy is intended to achieve data saturation within each stakeholder group for implementation outcomes framed by the PRISM and RE-AIM frameworks [67]. Recruitment will continue until thematic saturation is reached.

As this is a pilot randomized controlled trial using a hybrid type 2 implementation–effectiveness design [27], we aim to enroll 60 participants in total (30 per group), consistent with recommendations for hybrid pilot studies [68]. The primary objective of this pilot is to generate preliminary effect size estimates to inform sample size calculations for a future fully powered randomized controlled trial evaluating intervention effectiveness and to calculate the required number of implementation sites for a future fully powered Hybrid Type 2 trial.

#### Quantitative data analysis.

Implementation strategy outcomes will be analysed descriptively, using frequencies and proportions derived from PRISM/RE-AIM indicators. Fidelity to motivational interviewing (ITRS, CPMI) and self-determination theory (HCCQ) will similarly be summarised using descriptive statistics (means and standard deviations descriptively).

Quantitative outcomes, including physical activity, depressive symptoms, psychosocial measures, and physical fitness, will be analysed using linear mixed-effects models with F-tests to examine changes across time points (baseline, monthly assessments, post-intervention, and three-month follow-up). This approach accounts for repeated measurements within participants and accommodates missing data under the missing-at-random assumption. Effect sizes will be reported with 95% confidence intervals, and statistical significance will be set at p < 0.05. A statistician, blinded to group allocation and participant identity, will conduct all analyses using IBM SPSS Statistics 29.0.

In addition to primary outcome comparisons, mediation analyses will be conducted to explore whether changes in psychosocial factors, such as perceived social support and exercise self-efficacy mediate the effect of the BRIDGES intervention on depressive symptoms. We will use causal mediation analysis frameworks with bootstrapped confidence intervals to estimate indirect effects, while controlling for baseline values and relevant socio-demographic covariates. This will help elucidate potential mechanisms through which increased physical activity may improve mental health outcomes. Furthermore, clinical outcomes including re-hospitalisation and psychological relapse will be evaluated using logistic regression models, with odds ratios quantifying the association between intervention exposure (BRIDGES vs. usual care) and these outcomes. These analyses provide insight into both the effectiveness of the intervention and its potential real-world impact on clinical trajectories.

#### Qualitative data analysis.

Qualitative data collected through semi-structured interviews with adolescents, clinicians, and community partners will be analysed using the Qualitative Analysis Guide of Leuven (QUAGOL) [47], a systematic approach informed by principles of Grounded Theory [69]. The QUAGOL process begins with a preparatory “paper-and-pencil” phase, during which researchers iteratively read and summarize transcripts to develop preliminary conceptual insights. This is followed by systematic coding and analysis supported by NVivo 15 software (QSR International, Burlington, Massachusetts, USA). To enhance analytic rigor, multiple researchers will independently code transcripts, with discrepancies discussed and resolved through consensus. Qualitative findings will be reported in accordance with the Consolidated Criteria for Reporting Qualitative Research (COREQ) checklist [70]. These findings will complement quantitative results by elucidating contextual influences, implementation processes, and stakeholders’ experiences of the BRIDGES intervention. Together, this mixed-methods approach enables a comprehensive understanding of both intervention effectiveness and implementation dynamics.

### Data management

A Data Monitoring Committee (DMC) will be established for this trial, comprising two study investigators (AP and IB) and one external researcher (EM) who is not otherwise involved in trial conduct. The primary role of the DMC will be to oversee the integrity of the randomization process and ensure appropriate allocation concealment. The external member will be solely responsible for generating and managing the random allocation sequence and assigning participants to study groups. The DMC will also monitor overall study conduct, including adherence to the protocol and participant safety. No conflicts of interest are declared by any DMC member. Trial conduct will be reviewed monthly by the investigators, including verification of informed consent, adherence to the protocol, and completeness and accuracy of collected data. Given the low-risk nature of the intervention and small sample size, no additional formal monitoring visits are planned. This study will collect six types of data: informed assent and consent forms, socio-demographic information, questionnaires, clinical data including diagnoses, medication use, and fitness assessments, and interviews that will be audio-recorded, transcribed, and analysed using NVivo 15 (QSR International, Burlington, Massachusetts, USA). All participant data will be pseudonymized at the point of collection, with unique study identifiers replacing personal information. The master code linking participants’ identities to their study IDs will be stored separately in a secure, encrypted file accessible only to the principal investigator. Electronic data will be stored on institutional servers with secure login credentials, encrypted transmission, and regular backups, while hard-copy documents will be kept in locked filing cabinets within secure research offices. Access to identifiable data will be restricted to authorized personnel, including the principal investigator, designated research staff, and regulatory or funding auditors as required. Data collection and management will be conducted using secure, GDPR-compliant software platforms with audit trails to track all entries, modifications, or deletions. Automated and manual data quality checks will be performed regularly, and any discrepancies will be reviewed and resolved by the research team. Continuous monitoring will ensure adherence to the study protocol and maintain high data integrity. Data will be retained for 20 years following study completion, in accordance with institutional, ethical, and national requirements, after which all records will be securely destroyed or permanently de-identified. Pseudonymized datasets may be used for secondary analyses related to adolescent mental health, physical activity, or implementation science, provided such analyses are approved by the institutional ethics committee and align with participants’ original consent. Any sharing of de-identified datasets with external collaborators will be governed by formal data-sharing agreements to ensure confidentiality, ethical use, and compliance with legal regulations.

### Dissemination

Study findings will be communicated to multiple audiences. Participants and their caregivers will receive a plain-language summary of results at the conclusion of the study, while clinicians and community stakeholders will be engaged through presentations, workshops, and summary reports tailored to their professional roles. Results will also be submitted for publication in peer-reviewed journals and presented at national and international conferences. Dissemination will specifically highlight lessons for integrating co-designed, need-supportive physical activity interventions into outpatient adolescent mental health services, supporting evidence-based policy and clinical practice.

### Post-trial care

Following trial completion, participants assigned to the waitlist control group will be offered the opportunity to participate in the BRIDGES intervention, ensuring equitable access. All participants, regardless of study arm, will continue to receive standard care from their treating clinicians throughout and after the study. Any clinical issues identified during study assessments, including mental health deterioration or physical health concerns, will be promptly communicated to the participant’s clinician for appropriate follow-up and management.

## Expected results and discussion

This pilot hybrid type 2 randomized controlled trial will provide preliminary evidence on the implementation and effectiveness and of the BRIDGES intervention, a co-designed and theory-informed physical activity programme for adolescents with depression receiving outpatient care. By assessing both clinical outcomes and implementation processes, the study will generate insights into the intervention’s potential effectiveness and its feasibility for integration into routine clinical practice.

Implementation will be evaluated using the PRISM and RE-AIM frameworks [28,29] to identify factors influencing uptake, fidelity, acceptability and sustainability of the intervention. Barriers and facilitators at individual, organisational and community levels will be examined, including practical challenges related to scheduling, staffing and transition to community-based physical activity. Perspectives of adolescents, caregivers, clinicians and community partners will be collected to inform future optimisation and integration of the intervention within outpatient mental health care.

The pilot design, including recruitment in small waves, will allow assessment of feasibility for future trials, including recruitment rates, participant adherence, and appropriateness of outcome measures. Effect size estimates from this study will inform sample size calculations for fully powered RCTs, while qualitative data will provide a nuanced understanding of real-world implementation considerations.

We anticipate that participation in the BRIDGES program will lead to increased physical activity levels and reductions in depressive symptoms, consistent with existing evidence supporting structured, autonomy-supportive interventions for adolescents [17–21]. Beyond these primary outcomes, the study will explore secondary outcomes, including social support and exercise self-efficacy, which are hypothesized to mediate engagement in physical activity and broader mental health benefits [14,17,21,24].

We also expect improvements in cardiorespiratory fitness over time in the BRIDGES group compared with the waitlist control [71], while acknowledging potential heterogeneity in outcomes due to variation in the specific physical activities chosen by participants. If our findings demonstrate that fostering social connection, supporting autonomy of choice, and enhancing motivation, grounded in self-determination theory, can improve cardiorespiratory fitness, this would provide strong support for designing physical activity interventions that address low physical fitness in adolescent mental health populations, emphasizing the role of autonomous motivation [11,19,72].

This experimental intervention does not strictly adhere to the WHO’s evidence-based recommendation for adolescent physical activity; an average of 60 minutes per day of moderate-to-vigorous intensity aerobic activity, including muscle-strengthening exercises at least three times per week [30]. Instead, it addresses physical inactivity among adolescents with depression through a complementary approach within mental health care, combining structured evidence-based techniques with co-design principles that support autonomy and individualisation [8,73].

Insights from the pilot study will be particularly relevant for planning scalable interventions in outpatient settings, where engagement with physical activity is often lower than in structured inpatient programs [4]. Furthermore, documenting the transition to community-based physical activity opportunities may inform sustainable models for long-term engagement, bridging the gap between clinical care and community and local participation.

Beyond clinical practice, the findings are expected to have broader implications for policy and community mental health initiatives. Findings from the BRIDGES pilot may inform the integration of structured, autonomy-supportive physical activity programmes into outpatient mental health services and guide resource allocation for adolescent health promotion. Insights into strategies for transition to community-based physical activity may support collaboration between mental health services and local sports or youth organisations, promoting sustained participation. This study may contribute to the development of integrated care approaches linking clinical treatment with community-based physical activity for adolescents with depression.

Several limitations of the study are a-priori acknowledged. Integrating the RE-AIM framework within a randomized controlled trial design may constrain the ability to fully evaluate the BRIDGES intervention in real-world settings, as the structured trial procedures could limit ecological validity. Some potential participants may decline enrollment due to aspects of the study design, such as randomization or assignment to a waitlist control, which may reduce representativeness. Additionally, because the intervention represents a novel approach both within outpatient mental health care and in community-based physical activity settings, the attitudes and engagement of caregivers, coaches, and other stakeholders constitute a dynamic, evolving process. Variations in staff acceptance and the broader organizational climate may influence participant recruitment, adherence, and engagement, potentially resulting in heterogeneous outcomes over the course of implementation.

Throughout the protocol, we have adhered to SPIRIT 25 guidelines [26], ensuring comprehensive reporting of trial design, participant selection, interventions, outcome measures, and planned analyses. This structured approach enhances transparency, reproducibility, and methodological rigor, facilitating peer review and supporting replication in future trials.

## Conclusion

The BRIDGES pilot trial is expected to generate comprehensive, mixed-methods evidence on the preliminary effectiveness, and implementation of a co-designed physical activity program for adolescents with depression in outpatient care. Findings will inform the design of future large-scale trials and guide the integration of structured, need-supportive physical activity interventions into routine mental health services. By explicitly documenting intervention components, outcome measures, recruitment strategies, and implementation processes in line with SPIRIT 25, this protocol provides a transparent and replicable framework for evaluating lifestyle interventions in real-world clinical settings. Ultimately, the study aims to generate actionable knowledge to optimize both clinical and implementation outcomes, contributing to improved mental and physical health for adolescents living with depression, while supporting sustainable community engagement and policy development.

## Supporting information

S1 Appendix ClinicalTrials.gov document. (PDF)

S2 Appendix SPIRIT 25 guidelines. (PDF)

S3 Appendix Approved protocol by Ethic Committee. (PDF)
