# Supplementary material for: Bridging the gap: Implementing BRIDGES (Brussels Resilience and Inclusion for Depression - Guidance, Exercise and Support) to shape adolescent outpatient mental health care. A pilot randomized controlled study protocol
Source: PLoS One. 2026 Oct 27;21(10):e0360292. doi: 10.1371/journal.pone.0360292 (PMC13712597; doi:10.1371/journal.pone.0360292)
Supplement: Supplementary file 1 [file pone.0360292.s001.pdf]

ClinicalTrials.gov PRS
Protocol Registration and Results System

ClinicalTrials.gov Protocol Registration and Results System (PRS) Receipt
Release Date: June 16, 2026

ClinicalTrials.gov ID: NCT07422909

Study Identification

Unique Protocol ID: CCEMHA

Brief Title: Bridging the Gap: Implementing BRIDGES (CCEMHA)

Official Title: Bridging the Gap: Implementing BRIDGES (Brussels Resilience and Inclusion for Depression - Guidance, Exercise and Support) to Shape Adolescent Outpatient Mental Health Care. A Randomized Control Study Protocol

Secondary IDs: JRDIC-2 a-b-c [Grantor or Funder: Innoviris]

Study Status

Record Verification: June 2026

Overall Status: Enrolling by invitation

Study Start: March 1, 2026 [Actual]

Primary Completion: June 30, 2027 [Anticipated]

Study Completion: June 30, 2027 [Anticipated]

Sponsor/Collaborators

Sponsor: Université Catholique de Louvain

Responsible Party: Sponsor

Collaborators: KU Leuven

Oversight

U.S. FDA-regulated Drug: No

U.S. FDA-regulated Device: No

U.S. FDA IND/IDE: No

Human Subjects Review: Board Status: Approved
Approval Number: BE 4032024000098
Board Name: Comité Ethique Hospitalo-Facultaire Saint-Luc
Board Affiliation: UCLouvain
Phone:
Email: commission.ethique-saintluc@uclouvain.be
Address:

Promenade de l'Alma 51 bte B1.43.03 -1200 Bruxelles

Data Monitoring: Yes

Study Description

Brief Summary: Together with teenagers, we have developed a new approach: BRIDGES, which stands for “In Brussels, Resilience and Inclusion for Depression through Guidance, Exercise, and Support.” BRIDGES aims to help young people with depression get moving again in their daily lives, at their own pace and in ways that are good for them.

Detailed Description: Even though physical activity is recognized for its mental and physical health benefits, we find that adolescents going through a difficult psychological period often engage in very little of it. Current physical activity offerings do not always take into account their specific needs, preferences, or daily realities. That is why we have co-created a new approach with adolescents: BRIDGES, which stands for “In Brussels, Resilience and Inclusion for Depression through Guidance, Exercise, and Support.” This project includes 18 30-minute sessions over 3 months with a physical therapist specializing in mental health, including individual follow-ups with advice and discussions, as well as putting you in touch with sports clubs or associations of your choice. The goal is to help you get moving again in your daily life, at your own pace and in a way that makes you feel good.

This study has a waiting list control group, so young people can either participate immediately or wait 6 months to participate.

Conditions

Conditions: Inactive People
Depression

Keywords: Depression
Physical Activity
Adolescents
Community-level
Outpatient
Physiotherapy
Inactive
psychiatry

Study Design

Study Type: Interventional

Primary Purpose: Treatment

Study Phase: N/A

Interventional Study Model: Parallel Assignment
This study will use a hybrid type 2 effectiveness–implementation randomized controlled trial design, allowing simultaneous evaluation of the BRIDGES intervention’s impact on adolescents and the processes supporting its uptake in routine outpatient care. Implementation will be guided by the Practical, Robust Implementation and Sustainability Model (PRISM) and the RE-AIM framework, which together provide a structured approach to assess how the intervention is delivered, adopted, and sustained across outpatient and community-based settings. This dual perspective enables a comprehensive understanding of both the intervention’s effects and the factors influencing its successful integration into real-world clinical practice.

Number of Arms: 2

Masking: None (Open Label)

	Person in charge of randomisation Stastitician in charge of analysis
Allocation:	Randomized
Enrollment:	60 [Anticipated]

Arms and Interventions

Arms	Assigned Interventions
Experimental: The BRIDGES intervention<br>The BRIDGES intervention comprises 18 individualized sessions delivered over 12 weeks, combining physiotherapist-led counselling with structured support to transition into community-based physical activity.<br><br>Motivational interviewing techniques facilitate collaborative, non-judgmental dialogue, helping adolescents resolve ambivalence about physical activity. Through reflective listening and open-ended questions, physiotherapists guide participants to explore their values, goals, and motivations, fostering a sense of ownership over the behaviour change process. Drawing on the self-determination theory, counselling targets three key psychological needs.Relevant opportunities from the AREA+ network, including local sports clubs and initiatives, are introduced in ways that align with adolescents' interests and intrinsic motivation. All participating clubs and associations are carefully selected.	Behavioral: The BRIDGES (In Brussels, Resilience and Inclusion for Depression through Guidance, Exercise and Support) intervention<br>Described above
No Intervention: Waiting-list<br>Accordingly, the comparator group will be a waitlist control receiving usual care, allowing evaluation of the added benefit of the intervention within the existing care context. The usual standard outpatient care, which may include psychotherapy, medication management, or other usual clinical services. No structured physical activity intervention is provided.	

Outcome Measures

Primary Outcome Measure:

1. Depressive symptoms
   Patient Health Questionnaire-9 modified for Adolescents (PHQ-A) The PHQ-A is a brief instrument modified to adolescents used to assess the severity of depression (41). It contains 9 items that are on a scale from 0 to 3, with the total score ranging from 0 to 27.

   [Time Frame: Throughout the entire study, during approximately 15 months]
2. Physical activity levels
   Physical Activity Vital Sign (PAVS) The PAVS is a screening tool used to assess patients' physical activity levels (37). It consists of two questions: (a) on average, how many days per week do you engage in moderate to vigorous physical activity, and (b) on those days, how many minutes do you typically spend participating in moderate to vigorous physical activity? The product of these responses (days × minutes) provides an estimate of the patient's average weekly minutes of physical activity in moderate to vigorous physical activity.

   [Time Frame: Throughout the entire study, during approximately 15 months]

Secondary Outcome Measure:

3. Depressive symptoms (larger evaluation)

Beck Depression Index (BDI II) The BDI-II captures specific behavioral manifestations of depression. It is a 21-item self-report inventory, with each item scored from 0 to 3 based on four statements reflecting symptom severity, yielding a total score ranging from 0 to 63.

[Time Frame: Throughout the entire study, during approximately 15 months]

4. Multidimensional Scale of Perceived Social Support (MSPSS)
   The Multidimensional Scale of Perceived Social Support (MSPSS) is a widely used self-report instrument designed to assess an individual's perception of social support from different sources (49). The scale comprises 12 items, each rated on a 7-point Likert scale ranging from a score of 7 to 84.

   [Time Frame: Throughout the entire study, during approximately 15 months]

5. Adapted Exercise self-efficacy (SEE)
   Exercise self-efficacy (SEE) is a key determinant of intention formation and engagement in physical activity. In the original SEE, respondents rate their confidence in exercising three times per week for 20 minutes. For the present study, the scale was adapted so that the target behavior reflects an individualized exercise goal defined by each adolescent, that is, the type of physical activity they wish to perform.

   [Time Frame: Throughout the entire study, during approximately 15 months]

6. Astrand Rhyming test (ART)
   Astrand Rhyming test (ART) ART is a single-stage cycle ergometer test, to measure the CRF fitness levels (assessed as an estimation of VO2 max)

   [Time Frame: Throughout the entire study, during approximately 15 months]

7. Waist-to-height ratio (WHtR)
   Waist-to-height ratio (WHtR) WHtR is calculated as waist circumference (cm) divided by height (cm). Waist circumference will be measured in standing position, midway between the lowest border of the rib cage and the superior border of the iliac crest, at the end of a normal expiration.

   [Time Frame: Throughout the entire study, during approximately 15 months]

8. Motivational interviewing fidelity
   Fidelity to motivational interviewing (MI) will be assessed using a combination of observer- and participant-reported measures. First, adherence to MI principles will be evaluated using the Independent Tape Rater Scale (ITRS), which is recommended for effectiveness studies

   [Time Frame: Throughout the entire study, during approximately 15 months]

9. Client Perception of Motivational Interviewing
   the Client Perception of Motivational Interviewing (CPMI) scale captures participants' experiences of MI-consistent communication during sessions. This combined approach enables assessment of both the technical and relational components of MI fidelity while remaining feasible within the constraints of a pilot implementation study.

   [Time Frame: Throughout the entire study, during approximately 15 months]

10. The Health Care Climate Questionnaire
    Adolescents will complete the Health Care Climate Questionnaire (HCCQ) (61) to evaluate perceived autonomy support provided by the physiotherapist. In addition, brief post-session feedback forms will invite participants to rate the extent to which they felt understood, empowered, and supported in setting their own goals—key indicators of SDT-congruent delivery.

    [Time Frame: Throughout the entire study, during approximately 15 months]

11. Logbook
    the physiotherapist will fill out a logbook with their participation describing their presence, the context, the mechanisms at work, and clinical observations on the adolescents and their participation in physical activities

    [Time Frame: Throughout the entire study, during approximately 15 months]

Other Pre-specified Outcome Measures:

12. Implementation outcomes
    For the implementation assessment, PRISM will provide the overarching conceptual structure to examine organizational, individual, and system-level factors that influence delivery, uptake, and sustainability of the intervention (26). Within this framework, the RE-AIM dimensions (Reach, Effectiveness, Adoption, Implementation, and

Maintenance) will be systematically applied (27). Reach will assess the proportion and representativeness of adolescents. Effectiveness will evaluate changes in physical activity, depressive symptoms, and secondary outcomes. Adoption will explore barriers and facilitators to participation and delivery from the perspectives of adolescents, clinicians, and community partners. Implementation will monitor fidelity to motivational interviewing and self-determination theory principles. Maintenance will evaluate the potential for long-term integration of the intervention. Semi-structured interviews guided by PRISM domains will complement quantitative measures.

[Time Frame: Throughout the entire study, during approximately 15 months]

Eligibility

Minimum Age: 12 Years

Maximum Age: 19 Years

Sex: All

Gender Based: No

Accepts Healthy Volunteers: No

Criteria: Inclusion Criteria:

- All adolescents aged 12 to 19 with a diagnosis of depression who are receiving outpatient care through AREA+
- voluntary participation and accepting the randomisation
- being admitted at AREA+
- being able to stay focused for 30 minutes
- having a good knowledge of French
- physically inactive defined as not meeting the PA guidelines from World Health Organisation (28) following the corresponding age (i.e. at least 420 min of moderate to vigorous physical activity (MVPA) /week for those aged 12-17 and at least 150 min of MVPA /week for those aged 18 and 19 years
- living in Brussels

Exclusion Criteria:

- intellectual disability (defined as an intelligence quotient < 70)
- participants with serious medical problems or medical precautions prohibiting higher-intensity exercise (e.g., severe metabolic disorders, oncologic disease, unstable diabetes) according to the recommendations of the American College of Sports Medicine.

Contacts/Locations

Central Contact Person: Arnaud Philippot, PhD
Telephone: +32494827666
Email: arnaud.philippot@kuleuven.be

Central Contact Backup:

Study Officials: Arnaud Philippot, PhD
Study Principal Investigator
UCLouvain and KU Leuven

Marc Francaux, PhD
Study Director
Université Catholique de Louvain

Locations: **Belgium**
AREA+, Epsylon

Brussels, Uccle, Belgium, 1180
Contact: Vincent Dubois, Pr. and PhD +32477325890
v.dubois@epsylon.be

IPDSharing

Plan to Share IPD: No

References

Citations:

Philippot, A., Coppens, M., Van Damme, T., Sluys, C., Francaux, M., Dubois, V., & Vancampfort, D. (2026). Combining the behavior change wheel framework with a co-design approach to develop an outpatient physical activity intervention for adolescents with depression: A blueprint. Manuscript accepted for publication.

Miller, W. R., & Rollnick, S. (2012). Motivational interviewing: Helping people change. Guilford press.

Philippot A, Dubois V, Lambrechts K, Grogna D, Robert A, Jonckheer U, Chakib W, Beine A, Bleyenheuft Y, De Volder AG. Impact of physical exercise on depression and anxiety in adolescent inpatients: A randomized controlled trial. J Affect Disord. 2022 Mar 15;301:145-153. doi: 10.1016/j.jad.2022.01.011. Epub 2022 Jan 7. PubMed 35007642

Links:

Available IPD/Information:
