# Supplementary material for: Bridging the gap: Implementing BRIDGES (Brussels Resilience and Inclusion for Depression - Guidance, Exercise and Support) to shape adolescent outpatient mental health care. A pilot randomized controlled study protocol
Source: PLoS One. 2026 Oct 27;21(10):e0360292. doi: 10.1371/journal.pone.0360292 (PMC13712597; doi:10.1371/journal.pone.0360292)
Supplement: Supplementary file 3 [file pone.0360292.s003.pdf]

Protocol Title

Short title	Co-created exercise therapy in outpatient treatment in adolescents with mental disorders
Acronym / Protocol code	CCEMHA
Protocol version and date	First amendment 26/11/2025
Sponsor	UCLouvain<br>Belgium
Financial/Material support	Innoviris Brussels
Coordinating Investigator	Marc Francaux, marc.francaux@uclouvain.be

The information contained in this document is the property of the Sponsor/Co-ordinating/Principal Investigator and may not be reproduced, published or disclosed to others without written authorization of the Sponsor/Co-ordinating Principal Investigator.

Version History

Version	Approval Date		Changes
1.0	03/12/2024	Original	
2.0		Amendment	
3.0		Amendment	
4.0		Amendment	

1. Signature page

SPONSOR REPRESENTATIVE – COORDINATING INVESTIGATOR

Name	Signature	Date
Marc Francaux	[redacted]	19 july 2024

SITE PRINCIPAL INVESTIGATOR

I agree to conduct this study in accordance with the design and specific provisions of this protocol and will only make changes in the protocol after notifying the sponsor.

I understand that I may terminate or suspend enrolment of the study at any time if it becomes necessary to protect the best interests of the study subjects.

I agree to personally conduct or supervise this study and to ensure that all associates, colleagues, and employees assisting in the conduct of this study are informed about their obligations in meeting these commitments.

I will conduct the study in accordance with the protocol, Good Clinical Practice, the Declaration of Helsinki, and the moral, ethical and scientific principles that justify medical research. The study will be conducted in accordance with all relevant laws and regulations relating to clinical experimentation and the protection of patients.

I will ensure that the requirements relating to Ethics Committee review and approval are met.

I agree to maintain adequate and accurate records and to make those records available for audit and inspection in accordance with relevant regulatory requirements including the provision of direct access to data and source documents.

I agree to promptly report to the Ethics Committee any changes in the research activity and all unanticipated problems involving risks to human subjects or others. Additionally, I will not make any changes in the research without Ethics Committee approval, except where necessary to ensure the safety of study participants.

Name	Signature	Date
Philippot Arnaud	[redacted]	19 july 2024

2. Protocol synopsis

Title of Study : Co-created exercise therapy in outpatient treatment in adolescents with mental disorders

Acronym / Protocol code: CCEMHA

Sponsor: UCLouvain

Coordinating investigator: Marc Francaux

Study centre(s) and site(s) principal investigator(s): AREA+, Epsylon, chaussée de Saint-Job 294, Uccle and at the Exercise Physiology Lab, 1 Place Pierre de Coubertin, 1348 Louvain-la-Neuve

Pathology: Mental disorders

Rationale: Mental disorders are the main cause of illness and disability among adolescents aged 10-19. A lack of appropriate mental health care in adolescents is an important reason for persistence of residual symptoms and relapse. In addition, mental illness is a risk factor for physical co-morbidities. While exercise has shown promising results as a complementary treatment modality in reducing the mental and physical health burden in adolescents, in outpatient settings and real-world environments the evidence is lacking. Consequently, in this study, we will co-create an exercise intervention and evaluate it within a pilot randomised controlled, with the dual objective of assessing its preliminary effectiveness on both mental and physical health outcomes and examining key implementation parameters.

Objectives:
Primary

The main objective of this project is to co-create and evaluate an exercise-based post-hospitalization intervention as an outpatient treatment on depressive symptoms in adolescents with mental disorders. We will collect experiences from adolescents and staff on the pilot co-creation process

-Secondary

We will assess the effectiveness of a co-created exercise intervention (physical activity counseling and transition to physical activity community-level initiatives) on mental symptoms and physical outcomes, while simultaneously evaluating key implementation measures with a pilot randomized controlled trial.

Endpoints
Primary

<u>Psychological variable:</u>

Depressive symptoms by the Patient Health Questionnaire – 9 – adolescents version (PHQ-9M)
Physical Activity Vital Sign (PAVS) to measure physical activity levels

Secondary

<u>Physical variables</u>
Estimated VO_2max (ml/min/kg) measured with the Astrand-Rhyming test (ART)
Cardio-vascular risk by the waist-to-height ratio (weight in meter divided by Height in meter)

<u>Psychological symptoms:</u>

-The Beck Depression Index second version (BDI II)

-The perceived self-efficacy for exercise by the adapted Self-Efficacy for Exercise Scale (SEE)[1]
-Multidimensional Scale of Perceived Social Support (MSPSS)

Implementation variables<br>- Semi-structured interview based on Practical, Robust Implementation and Sustainability Model (PRISM) to evaluate the Reach, Effectiveness, Adoption, Implementation, and Maintenance (RE–AIM)
**Study Design:** randomized controlled trial
**Number of patients:** 60 (control group (30) + co-created exercise group (30)), adolescents (12-19 years)
**Main criteria for inclusion (inclusion/exclusion criteria)**<br>-Inclusion criteria are: (a) aged between 12 and 19 years, (b) being admitted at AREA+, (c) being able to stay focused on a task for 30 minutes based on the medical interview at the intake (d) having a proficient knowledge of French to allow filling out the questionnaires and understand instructions, (e) inactive status, defined as not meeting the PA guidelines from World Health Organisation following the corresponding age (i.e. at least 420 min of Moderate and Vigorous Physical Activity (MVPA) /week for those aged 12-17 and at least 150 min of MVPA /week for those aged 18 and 19 years, (f) living in Brussels.<br>-Exclusion criteria are (a) planned hospitalisation during the intervention (b) physical disability preventing administration of standardised tests (e.g., inability to sit on a bicycle); (c) intellectual disability (defined as an intelligence quotient < 70); and (d) participants with serious medical problems or medical precautions prohibiting higher-intensity exercise (e.g., severe metabolic disorders, oncologic disease, unstable diabetes) according to the recommendations of the American College of Sports Medicine (1)which are implemented within the clinical setting; for the randomized controlled trial only, (e) being pregnant. A general practitioner will review the medical conditions and precautions for each patient to this end.
**Total study duration for the RCT:**<br>- date of planned first enrolment: 1/03/2026<br>- date of planned last completed: 1/08/2027
**Study procedures:** Schedule of assessments – Study Flowchart
**Statistical Considerations**: Linear mixed model with F-test
**Publication (reference)**<br>-Philippot A, Dubois V, Lambrechts K, Grogna D, Robert A, Jonckheer U, et al. Impact of physical exercise on depression and anxiety in adolescent inpatients: A randomized controlled trial. Journal of affective disorders. 2022.<br>-Vancampfort, D., Firth, J., Correll, C. U., Solmi, M., Siskind, D., De Hert, M., ... & Stubbs, B. (2019). The impact of pharmacological and non-pharmacological interventions to improve physical health outcomes in people with schizophrenia: a meta-review of meta-analyses of randomized controlled trials. World Psychiatry, 18(1), 53-66.<br>-Vancampfort, D., Mugisha, J., Byansi, P. K., Namutebi, H., Rosenbaum, S., Lukwata, H., & Ward, P. B. (2022). Mental contrasting and implementation of physical activity intentions in Ugandan primary care patients with mental health problems: A real-world intervention involving support partners. Psychiatry Research, 307, 114335.<br>-Van Damme, T., Simons, J., Sabbe, B., & Van West, D. (2015). Motor abilities of children and adolescents with a psychiatric condition: A systematic literature review. World journal of psychiatry, 5(3), 315.<br>-Vancampfort, D., Probst, M., Daenen, A., Van Damme, T., De Hert, M., Rosenbaum, S., & Bruyninckx, D. (2016). Impact of antipsychotic medication on physical activity and physical fitness in adolescents: An exploratory study. Psychiatry Research, 242, 192-197.

3. SCHEDULE OF ACTIVITIES

Flow-chart 3 : Pilot randomized controlled trial

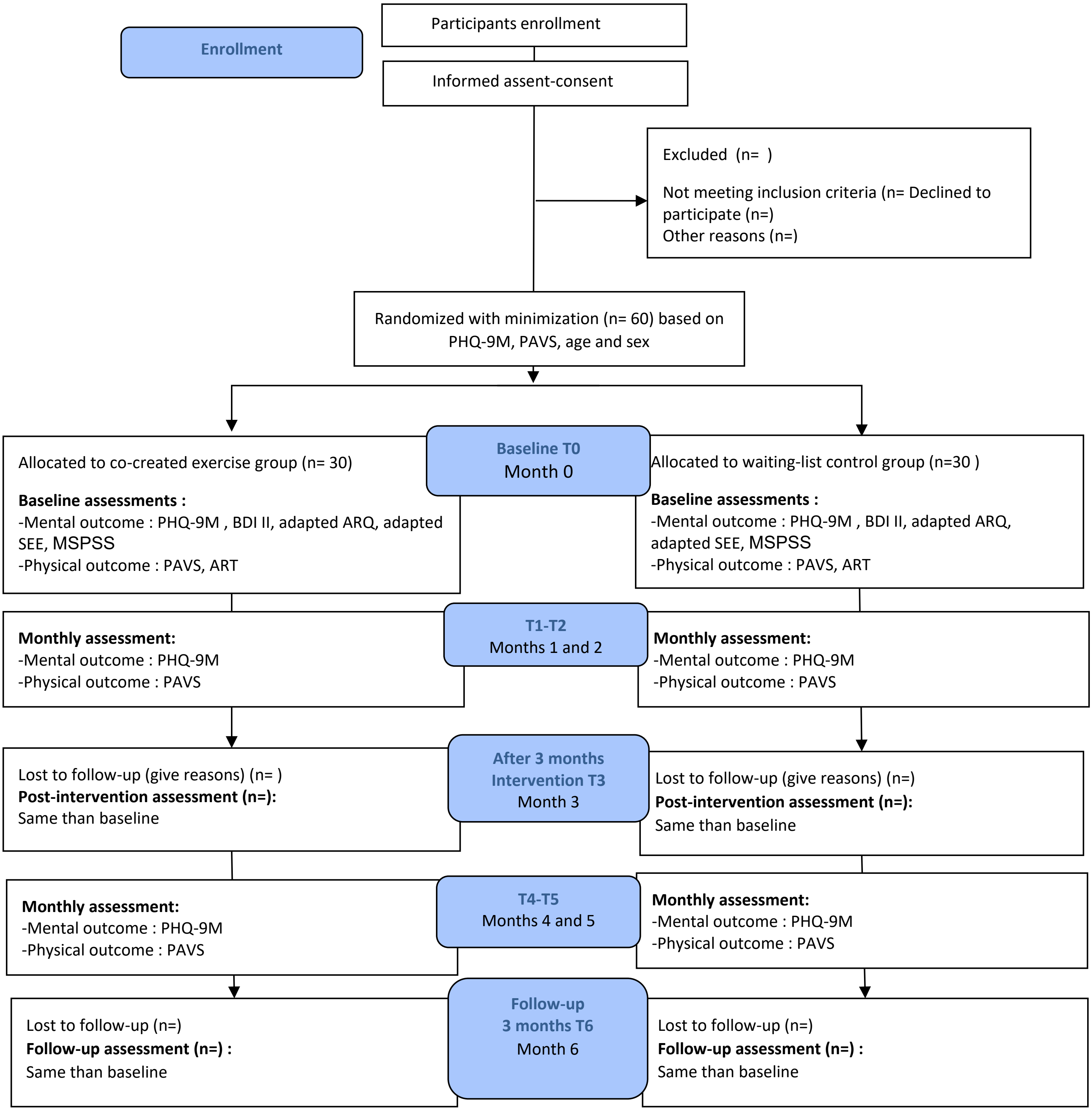

Table of content

1. SIGNATURE PAGE 3

2. PROTOCOL SYNOPSIS 4

3. SCHEDULE OF ACTIVITIES 7

4. LIST OF ABBREVIATIONS AND DEFINITIONS 9

5. ETHICS 9

6. OBJECTIVES 10

PRIMARY 10
SECONDARY 10

7. ENDPOINTS 10

8. BACKGROUND INFORMATION AND SCIENTIFIC RATIONALE 20

MEDICAL BACKGROUND 20
RATIONALE 21

9. INVESTIGATIONAL PLAN 23

DESIGN 23
STRATEGIES FOR PARTICIPANT RECRUITMENT 27
PARTICIPANTS ELIGIBILITY 20
START OF THE STUDY 30

10. STUDY INTERVENTIONS AND PROCEDURES 30

METHOD OF ASSIGNING PARTICIPANT TO INTERVENTIONS GROUPS 34
SAMPLE LAB COLLECTION 34
PROTOCOL DEVIATIONS 35
DATA QUALITY ASSURANCE 35
STATISTICAL ANALYSIS 36
PROTOCOL AMENDEMENTS 38
DATA MANAGEMENT RESPONSIBILITIES 39

11. FINANCE AND INSURANCE 41

12. END OF STUDY 41

FOR AN INDIVIDUAL SUBJECT 41
FOR THE WHOLE STUDY 41

13. DISSEMINATION OF RESULTS AND PUBLICATION POLICY 41

14. ARCHIVING 41

15. STUDY REPORT 42

16. LITERATURE REFERENCES 42

17. APPENDIX 47

4. List of abbreviations and definitions

Exercise is "physical activity that is planned, structured, and repetitive and has as a final or intermediate objective to improve or maintain physical fitness" (2).

Co-creation is described as "bringing everyone around the table to make the most of each other's knowledge". Co-creation is the process of involving stakeholders in developing interventions, defining directions and goals, and solving problems together (3). Co-creation models have a high potential for societal impact but are highly dependent on key principles for success (4).

In the context of health services, **implementation** refers to the integrating of evidence-based interventions into real-world clinical or community settings in a manner that ensures their feasibility, fidelity, acceptability, and sustainability. It encompasses the strategies, contextual determinants, and organizational mechanisms that shape how an intervention is adopted, delivered, and maintained over time (5, 6).

5. Ethics

- This protocol, any protocol amendments, informed consent form and other relevant documents (eg. recruitment advertisements) will be submitted to the Ethics Committee (EC) for formal approval to conduct the study. The decision of the EC concerning the conduct of the study will be made in writing to the sponsor. All correspondence with the Ethics Committee will be retained in the Investigator File.

- The study will be conducted in accordance with legal and regulatory requirements (Belgian law of 7 May 2004, Belgian law for Patient rights 22 August 2002, Private life GDPR 2018), as well as the Guidelines for Good Clinical Practice (International Conference on Harmonization 1996), and the last version of Declaration of Helsinki (World Medical Association).

- All subjects for this study will be provided a consent form describing this study and providing sufficient information for subjects to make an informed decision about their participation in this study. This consent form will be submitted with the protocol for review and approval by the EC. The formal consent of a subject, using the EC-approved consent form, will be obtained before that subject is submitted to any study procedure. This consent form must be signed by the subject or legally acceptable surrogate, and the investigator-designated research professional obtaining the consent. The written informed consent document should be prepared in the language of the potential patient population.

- The identity of the participant will remain kept confidential according to the General Data Protection Regulation of 27 April 2016 (in application on 25 May 2018), to the Belgian law of 30 July 2018 on the protection of natural persons with regard to the processing of personal data and the Belgian patient's right law (22 August 2002). Personal data will be coded. Subjects will not be identified by name or in any other recognizable way in any of the records, results or publications related to the experiment.

6. Objectives

Primary

The main objective of this project is to co-create and evaluate an exercise-based post-hospitalization intervention as an outpatient treatment on depressive symptoms in adolescents with mental disorders.

Secondary

We will assess the impact of co-created exercise intervention (physical activity counseling and transition to physical activity community-level initiatives) on a variety of psychological symptoms and physical outcomes, and implementation variables. In order to inform the intervention, we will a-priori investigate to what extent the physical fitness of adolescents with mental disorders is impaired compared to matched general population controls.

Endpoints

Randomized controlled trial

We will use a hybrid effectiveness-implementation design (type 2), which enable simultaneous examination of both the implementation process and the effectiveness, particularly in relation to its uptake within real-world settings (7).

Primary effectiveness outcomes

For the effectiveness part of BRIDGES, quantitative outcomes will assess monthly changes from baseline to post-intervention and three-month follow-up (6 months in total) for PA levels and for depressive symptoms.

- The Physical Activity Vital Sign (PAVS) is a screening tool used to assess patients' physical activity levels (8). It consists of two questions: (1) on average, how many days per week do you engage in moderate to vigorous physical activity, and (2) on those days, how many minutes do you typically spend participating in moderate to vigorous physical activity? The product of these responses (days × minutes) provides an estimate of the patient's average weekly minutes of physical activity in moderate to vigorous physical activity. This screening tool is also sensitive to changes in mental health care settings (9) and able to identify physical inactivity associated with cardio-metabolic risk factors in youth with mental illness (10, 11).
- Patient Health Questionnaire-9 modified (PHQ-9M) is a brief instrument modified to adolescents used to assess the severity of depression (12). It contains 9 items that are on a scale from 0 to 3, with the total score ranging from 0 to 27. Adapted from the Diagnostic and Statistical Manual of Mental Disorders, Fourth Edition (DSM-IV), the PHQ-9M encompasses the same diagnostic symptom criteria outlined in the DSM-IV, including, the two cardinal signs of depression (anhedonia and depressed mood), cognitive symptoms and physical symptoms (13). The PHQ-9M is shorter than many other depression screening tools and can be self-administered. It's a valid and reliable assessment tool for the severity of depressive symptoms in adolescents with major depression disorder (12) and is sensitive to changes among adolescents in psychiatric setting with reliable change index of 7 (14).

Primary implementation outcomes

The implementation process will last throughout the intervention period, 12 months. For the implementation assessment, we will be guided by the Practical, Robust Implementation and Sustainability Model (PRISM) (15) to assess the Reach, Effectiveness, Adoption, Implementation, and Maintenance (RE–AIM) (16) of the physical activity counseling intervention, as well as the transition from mental health services to community-level, see table 1. Implementation research is recommended in development of lifestyle psychiatry treatment into real world setting (17-19).

Table 1. Evaluation of the implementation using RE-AIM framework

Concept	Measure
**R**each	Reach will be defined as the proportion participants who consent to participate in the intervention during the specified recruitment period. In addition to this participation rate, we will compare socio-demographic characteristics, e.g., age (years), sex, gender (females, males, others), school dropout duration, family structure (presence of two parents, only one and under guardian), duration of depression (months/years), lifetime of depression (n lived experience (s) of depression), socio-economic status (BIM status), co-occurring mental health difficulties/disorders (anxiety, ADHD, experience of psychosis, PTSD) and somatic/physical condition (diabetes, obesity, asthma, others) of those who consented versus those who declined participation to assess representativeness. All these data will be found in the medical file of participants, under assent and consent of data using for research purposes delivered by AREA+ setting hospital. Further, we will document the total number of outpatients screened for eligibility, reasons for ineligibility, and basic socio-demographic characteristics of those excluded. This will allow us to assess the potential for selection bias and the generalizability of the intervention.
**E**ffectiveness	Effectiveness will be evaluated both quantitatively and qualitatively. Primary outcomes include changes in physical activity levels and depressive symptoms, assessed using the PAVS and PHQ-9M, respectively as described previously. Secondary outcomes will include perceived changes in physical, mental, social well-being, usual care follow-up and potential discharge which will be explored qualitatively through brief semi-structured interviews conducted post-intervention and after follow-up. Adverse events and unintended consequences will also be documented to assess intervention safety.
**A**doption	Adoption will be assessed through qualitative interviews with patients after their participation and staff from the sport clubs and healthcare staff at the end of implementation phase (12 months). These interviews will explore perceived barriers and facilitators to participating in or delivering the physical activity counselling. Insights gained will help determine the acceptability and feasibility of the intervention from multiple stakeholder perspectives, and provide a better understanding of factors influencing uptake across individuals and clinical contexts.
**I**mplementatio n	Implementation will focus on fidelity to the intervention protocol. Specifically, we will monitor whether key components are delivered consistently, including: (1) initial physical activity assessment during the first evaluation (T0), (2) completion

	of the planned counselling sessions (3) ongoing participation in community-level at the follow-up and (4) lived experience of adolescents on the physical activity counseling sessions. For this fourth part, the MI consistent communication via CPMI scaleand perceived autonomy support from their physiotherapist via HCCQ. In addition, brief post-session feedback forms will ask participants to rate the extent to which they felt understood, empowered, and supported in setting their own goals—key indicators of SDT-congruent delivery. Fidelity data will be triangulated with qualitative feedback from both adolescents and physiotherapists to identify contextual influences on delivery, drift, and opportunities for reinforcement during training and supervision. Maintaining high fidelity to MI and SDT principles will be considered a core implementation outcome, as it directly relates to the mechanisms of action underpinning behavior change.<br>Deviations from the protocol, reasons for missed sessions, and adaptations made in clinical practice will also be recorded. This process evaluation will inform the degree to which the intervention was delivered as intended.
**M**aintenance	Maintenance will focus on the potential for long-term integration of the physical activity counselling program into routine clinical care. This will include assessing after 6 months whether program components are used beyond the study period, staff willingness and capacity to sustain delivery, and the extent to which the intervention aligns with existing workflows and institutional priorities. Follow-up interviews with staff and review of service delivery data (e.g. recorded physiotherapy sessions for outpatients with depression in the medical files) will support this assessment.

PAVS= Physical Activity Vital Sign
PHQ-9M= Patient Health Questionnaire-9 modified
CPMI= Client Perception of Motivational Interviewing
HCCQ = Health Care Climate Questionnaire
SDT= Self-Determination Theory
MI= Motivational Interview

Table 2. PRISM domains addressed in the semi-structured interviews

**PRISM domain**	**Participant groups**	**Examples of questions**
Implementation structure	Physiotherapist	- What ideas do you have for maximizing efficiency in the workflow for (a) the assessments, (b) the brief physical activity counseling intervention?
External environment	Clinicians and management	- I'm interested in learning about your impressions of the current climate about physical activity counseling as part of a holistic care for adolescents outpatients here. In your opinion, do you believe your setting has the capacity for incorporating an initiative like the one we executed in this pilot study?<br>- Considering the climate as well as the potential benefits and barriers of this initiative, what are your thoughts regarding the implementation of the intervention?

Clubs/assocations in community-level	Coaches /physical activity providers	- I would like to know your opinion on the integration of adolescents suffering from depression whom you have hosted. In your opinion, do you think that your institution and your staff have the capacity to implement an initiative such as the one we have carried out as part of this pilot study?
Intervention design	Patients	- What do you like or dislike about the current intervention?<br>- Could you give feedback or suggestions about potential adaptations you consider of added value?<br>- How do these sessions support your motivation?

Secondary outcome (baseline/ post-intervention/ post-follow-up)

For secondary outcomes, we will evaluate depression symptoms, resilience, social support and cardio-respiratory fitness at three times (baseline, post-intervention and after the follow-up period).

Effectiveness measures

Psychological symptoms

- **The Beck Depression Index** (BDI II) describes a specific behavioural manifestation of depression. BDI-II is 21-item self-report inventory graded from 0 to 3 by a series of 4 statements reflecting the degree of severity of the symptom. It's valid and reliable in adolescents with major depression (20, 21). While PHQ-9M is brief and time-efficient, BDI II is more complex for the emotional changes among adolescents (12). So, the combination of the two questionnaires conduct to a more comprehensively evaluation over time (20).
- **Adapted Adolescent Resilience Questionnaire** (adapted ARQ) is revised and shorter questionnaire comprises 49 items and 12 scales measuring resilience factors in the domains of self, family, peer, school and community (22). All scales have acceptable alpha coefficients. It offers a comprehensive and developmentally appropriate instrument to measure a young person's capacity to achieve positive outcomes despite life stressors. No data exist in clinical population of adolescents but remains the only one that was designed for adolescents.

The adapted ARQ is not currently available in French. Authorization for translation has been obtained from the original author, but approval from the Ethics Committee is also required. The questionnaire will only be administered to adolescents once the Ethics Committee has granted approval and the translated version has been finalized.

- **Multidimensional Scale of Perceived Social Support** (MSPSS) is a frequently used scale for evaluating the social support (23). Twelve-item ratings are made on a 7-point Likert-type scale divided into 3-subscale (significant other, family, and friends). It demonstrated high internal consistency in psychiatric outpatients (24) and in young people (23, 25).
- **The adapted Self-Efficacy for Exercise Scale** (SEE) contains 9 items on 11-point likert-type scale (26) and showed excellent internal consistency among adolescents (27). Self-efficacy was found to be a major instigating force in forming an intention to be active. The responses requested in the initial SEE were based on the original aim: "How confident are you right now that you could exercise three times per week for 20 minutes?". For this study, we adapted the

measure so that the aim reflects an ideal goal defined by the adolescents themselves: the exercise behavior they wish to perform. Despite this modification, the questionnaire will continue to use the standardized format of the original items.

Physical fitness

- **Astrand Rhyming test** (ART) is a single-stage cycle ergometer test, to measure the CRF fitness levels (assessed as an estimation of VO2 max). It is designed to elicit a steady-state heart rate over a 6-min period. The initial workload will be selected from the Astrand test loading wattage table, pedalling speed remains constant (60 revolutions per min) and heart rate (HR) is recorded with a Polar heart rate sensor at every minute interval. The workload is set during the first 2 min and maintained throughout the test. In the case that HR fails to achieve the target zone, the load is adjusted accordingly. HR and loading wattage are noted at the end of each minute, with a target goal of obtaining two consecutive HR values over 120 beats but below 170 beats per minute (bpm), within 5 bpm of each other, during the fifth and sixth minute of work. The protocol will be interrupted if threatening symptoms appear or when the HR reached 85% of age-predicted (220-age) maximum HR. (11).

- **Waist-to-height ratio** *(WHtR)* (15,16) is calculated as waist circumference (cm) divided by height (cm). Waist circumference will be measured in standing position, midway between the lowest border of the rib cage and the superior border of the iliac crest, at the end of a normal expiration. Three waist circumference measurements will be taken with a non-stretchable tape and the mean value is used in the WHtR calculation. The universal WHtR cut-off of 0.5 will be used to identify participants at increased cardiometabolic risk.

7. Background Information and Scientific Rationale

Medical Background

Mental disorders are the main cause of illness and disability among adolescents aged 10-19, leading to social exclusion, discrimination, educational difficulties, and stigma (WHO 2021). Having a mental disorder is also an important risk factor for reduced physical fitness (28, 29) which, in turn, is a risk factor for future chronic conditions such as cardiovascular diseases (30, 31) and, ultimately, premature mortality later in adulthood (32). Surprisingly, physical fitness levels have never been assessed using validated measures in adolescents with mental disorders. Nor have their physical fitness levels been compared to age-, sex- and health inequalities matched adolescents without a mental illness (31). In recent years, among mental disorders, mainly depression and anxiety rates increased significantly in Belgium, including in Brussels (33, 34). Nowadays 34% of teenagers aged 10-19 years are at risk of developing clinical depression (34), while for those who experienced depression there is a high risk for residual symptoms impairing quality of life and a particularly high risk of relapse (39%-72%) (35, 36). A lack of appropriate mental health care for depression in adolescents is an important reason for persistence of residual symptoms and relapse which ultimately could result in serious and long-term psychological, cognitive, social, behavioural, physical and educational impairments (37).
While exercise has shown promising results as a complementary treatment modality in reducing the mental health burden in adolescents, as supported by recent research and meta-analyses by the academic partners involved in this project (38-41), in outpatient settings and real-world environments the evidence is lacking. Moreover, dropout to treatment in general in adolescents is high and this might

be due to the fact that interventions are not tailored to the wishes and needs of the adolescents (40). To assure their compliance and efficacy of intervention, co-creation will be used as a user-centred participatory approach of research to optimise effectiveness and sustainability of the intervention. Co-creation process will be provided in using the Behavior Change Wheel and Co-creation Framework (42) and Self-determination Theory (43). The behaviour change wheel is a theoretical framework that ensures the scientific and systematic development of an intervention, while Self-determination Theory suggests that everyone has three fundamental psychological needs - autonomy, competence and relationship - that underpin growth and development. Although co-creation (44-46) and theoretical underpinning (47-49) are becoming more widespread in developing and evaluating mental health care interventions, reports of the process are scarce, and, to date no co-created exercise study has been conducted with adolescents with mental disorders. This project is therefore from an academic perspective very innovative. Finally, an exercise intervention (guided by the self-determination theory and behaviour change wheel framework) will be co-created. Mental, physical and implementation outcomes of this co-created intervention will be evaluated within a pilot Randomised Controlled Trial (RCT).

From a clinical perspective, the project has several strengths. Implementation of physical activity remains fragmented and poorly integrated into mental health routine care (17, 18) and this project answers to persistent implementation gaps between evidence and practice (19) and the need of connections between mental health care and community-level services (50, 51).

Rationale

Statement of the hypothesis

Two main hypotheses:

1. Adding exercise to outpatient treatment will lead to a greater reduction in depressive symptoms compared to treatment as usual only

Secondary hypotheses:

2. Co-created exercise will provide greater reduction in a wide range of psychological symptoms, improvements in physical outcomes, and will provide insights into barriers, facilitators, and contextual factors influencing its acceptability, its delivery, and its sustainability within outpatient routine care.
3. Based on previous research in other clinical populations (52), we hypothesize that there will be a moderate to high correlation between easy-to-use and laboratory tests and that easy-to-use tests will be feasible and reliable.

Description of the study population, disease, current standard of care

Adolescents, hospitalized in psychiatric units as in AREA+ could have only one but also multiple mental disorders (depression, anxiety, post-traumatic symptoms, disruptive behaviour, social withdrawal, bizarre thoughts or hallucinations, scarifications, etc.). It is essential that adolescents remain supported upon discharge to prevent relapses including comorbid symptoms of depression and anxiety and physical deconditioning (31, 53). A lack of appropriate mental health care for depression in adolescents is an important reason for persistence of residual symptoms and relapse which ultimately could result in serious and long-term psychological, cognitive, social, behavioural, physical and educational impairments (54). Several calls have been made to ensure access to appropriate mental and physical health care for adolescents developing mental disorders (31, 53), in particular at community level (55). In Brussels, after hospitalisation, the current standard of care is the psychotherapeutic and pharmacologic treatment in adolescents (56). An add-on potential non-pharmacological approach for reducing (comorbid) depression and/or anxiety symptoms, physical

health burden and preventing relapse is exercise therapy (57-59). However, no such post-hospitalisation initiatives exist in Brussels. In addition, physical activity providers in community-level could play a pivotal role in the transition between outpatient mental health care and community-level support (60-62). But there is a need for research evaluating if psychological -and trauma-informed physical activity training modules may be implemented among them.

A summary of the known and potential risks and benefits, including an assessment of the expected benefits and risks

We hypothesize that the co-created exercise intervention will reduce the physical and mental health burden of adolescents with mental health disorders. We hypothesize as well that the interventions will lead to reduced re-admission rates in psychiatric services and future admissions for somatic disorders such as cardiovascular diseases.

There are no main study-related risks. However

- Adherence for adolescents in mental health treatment is usually poor and they are at higher risk of drop out in research studies (63). In addition, exercise has less dropouts when the providers are exercise experts (64). Researchers who will carry out interventions will be physiotherapists and physical teachers. All assessments and tests will be conducted with researchers, who are trained in working with adolescents with mental health problems and in the assessments.
- Evaluation time can be long and recruitment is important to obtain required sample size. To encourage participation, we will provide feedback to the adolescents on their physical fitness status and formulate some recommendations, if they wish. The co-creation process will make it more attractive to adolescents following the intervention. In addition, to cover transport and participation costs, indemnities for the adolescents will also be given per completed evaluation time.
- To prevent any risk to perform a (sub)maximal test and during the co-created exercise intervention, the Physical Activity Readiness Questionnaire (PAR-Q) will be completed and a general practitioner will review the medical conditions and precautions for each patient to this end. To prevent possible nausea and uncomfortable physical sensations during the maximal effort tests, participants will be advised to eat a balanced meal and keep well hydrated two hours before the test, and to avoid eating a copious meal. If they do experience such sensations, they will be invited to spend some time recovering in a quiet place and will be offered access to the toilet if necessary.
- To prevent any risk of exercise-related injury, we always recommend warming up the whole body during exercise sessions and provide recommendations tailored to the participants' physical and mood level and knowledge of exercise. If an injury or an accident appear, the document “case report form, adverse cases” for no-severe cases will be completed and sent in the final report to Ethic Committee.
- The medical organization and care will be ensured by Alexandre Beine, MD, medical director and head of the psychiatric department of AREA+ and by Vincent Dubois, MD, PhD, professor of psychiatry at the Faculty of Medicine of the UCLouvain, managing director and general medical director of the ASBL Epsylon
- Expertise is available within the lab of KU Leuven that will participate with the post-doctorate candidate Arnaud Philippot, UCLouvain that will participate with a doctorate candidate and AREA+, Epsylon with a physiotherapist. These three people will be funded during three years of this project by Innoviris. The KU Leuven lab has a strong research expertise in evaluating exercise interventions for various mental health disorders including depression, anxiety, post-traumatic stress disorder and autism spectrum disorder and in assessing cardiorespiratory fitness of people with severe mental illness (65, 66). The UCLouvain exercise lab has acquired large experience and expertise in fitness condition among professional athletes and trainers but also populations with

various and complex pathologies, such as intensive care patients, people with diabetes and the elderly. Epsylon is a major player in the provision of psychiatric care in Brussels and covers all the modalities of mental health care. This study can take advantage of the ongoing prospective project in youth aged 12-16 years (internal funds from the KU Leuven: 3M180299, C14/18/097; 01.10.2018 to 30.09.2022 and an Aspirant FWO fellowship: M200112, 11M4722N; start 01.11.2021) and an ongoing study on physical activity and cardiorespiratory fitness in adolescents with autism spectrum disorder (Steunfonds Marguerite-Marie Delacroix : 3M200597). In the latter project also maximal incremental exercise tests are being performed. These projects are co-supervised by Prof. Marc Francaux and Prof. Louise Deldicque from UCLouvain as the main researchers with support from Prof. Davy Vancampfort, Prof. Tine Van Damme from KU Leuven.

8. Investigational plan

Design

Experimentation type
Pilot Randomized Controlled Trial (RCT)
Setting study
Chaussée de Saint-Job 296, 1180 Uccle for the co-creation process, RCT
Expected direct individual benefit
For mental and physical health of recruited adolescents
Randomization with minimization
RCT: We will include randomisation by minimization with criteria such as age, sex, level of depression symptoms. Based on the outcome on these tests, the participants will be allocated randomly. The randomization principles is more explained below.
Study groups
RCT: Treatment As Usual (TAU) group and TAU + exercise intervention group
Expected duration of the study
One year for the pilot RCT

Expected duration of subject participation:

Interventional study: 3 hours per participant

Co-creation process: 5 sessions of 2 hours comprised in a duration of 5 weeks

RCT: 18 physical activity counseling sessions of 30 minutes in a duration of 12 weeks + 3 months of follow-up

Training implementation: 3.5 hours of workshop

Methods for collecting data for assessment of study objectives:

<u>RCT:</u>

Participants, after their hospitalisation and in outpatient setting, will complete data through redcap system.

1. Visits in baseline (T0), post-intervention (T3), in follow-up (T6) in AREA+

In person, in a calm room, for adolescents from AREA+

- Data on demographic measures (age, sex, declared gender, medications, actual care, waist-to-height ratio)

- Mental health and physical measures via self-reports (PHQ-9M, PAVS, BDI-II, adapted ARQ, SEE, MSPSS) and the completion of the ART on a cyclo-ergometer
- In post-intervention (T3) for co-created intervention only: the adolescent perception of the motivational interviewing principles (CPMI) and their perceived autonomy support on the lived physical activity counselling sessions and (HCCQ) and Effectiveness variable in the RE-AIM framework through qualitative interviews on their on their lived experience (perceived changes in physical, mental, social well-being)
- In follow-up (T6) and co-created intervention only: Effectiveness, Adoption and Implementation variables in the RE-AIM framework through qualitative interviews guided by PRISM with the adolescents on their lived experience (perceived changes in physical, mental, social well-being), their barriers and their facilitators of the intervention.

2. Monthly evaluation (T2,T3,T4,T5)

Online, QR code sent, for adolescents from AREA+

- Depressive symptoms and physical activity levels measures via self-reports (PHQ-9M, PAVS) sent by QR on their phone number or mail following the preference through redcap system

3. Online Brief feedbacks in post- physical activity counseling session

Online, QR code sent, for adolescents from AREA+

- Forms on the extent to which they felt understood, empowered, and supported via QR code at each session of the intervention for each adolescent (18 sessions)

4. After 12-months intervention running

In person, for staffs in AREA+ and in community-level

- Adoption in the RE-AIM framework through qualitative interviews guided by PRISM will be assessed through qualitative interviews with staff from healthcare and the sport clubs at the end of implementation phase (12 months). Adolescents are not included at this stage.
- At the end of the 12-month intervention period, the *Maintenance* dimension of the RE-AIM framework will be assessed six months later. This will involve examining whether program components continue to be used beyond the study period, evaluating staff willingness and capacity to sustain delivery, and determining the extent to which the intervention aligns with existing workflows and institutional priorities.

Participants in the waitlist control group will immediately receive a FNAC voucher of 15 euros for their visits T0, T3, and T6. Adolescents in the experimental group will receive a voucher of 15 euros only in the follow-up period (T6) assessment. Participants will receive a €15 voucher as compensation for their attendance and the time required to complete this supplementary evaluation period. Individuals who discontinue participation or do not complete the evaluation will not be eligible to receive this compensation.

Studied population and number of planned patients

1. RCT: 30 inactive participants with depression per group from AREA+ in outpatient setting for a total of **60 adolescents (30+30). 12 expected stakeholders** (adolescents, caregivers and

managers of mental health care setting, coaches in community-level) for each variable in RE-AIM framework requesting a semi-structured interview.

Strategies for participant recruitment

Potential participants will be recruited from the inpatient or outpatient (RCT) psychiatric units of AREA+ Epsylon. The psychiatrist in AREA+, in charge of the care for the patient, will review whether the patient can be included (see inclusion and exclusion criteria). If eligible, the clinical and research assistant will invite the potential participant and discuss the information letter. All participants will provide their written informed assent and the written informed consent will be obtained from the parents/ legal representatives for minors before participation. In AREA+, there are 316 new patients per year in the units.

Potential matched peers for the control group in the interventional study will be recruited in local school in Brussels and social media.

The informed consent for parents and major participants and assent for minor participants have been available for the co-creation process and for the RCT in the submitted documents for the Ethic Committee.

Informed consent process

Information related to the study is provided to patients and their legal representative by the investigator during the consultation, according to the requirements pertaining to consent covered by ICH-GCP (E6). There are also informed they could withdraw their consent at any time during the study without any consequence and without providing a reason. This point is written in the informed consent/assent form.

Patients and their legal representative receive the patient information and consent/assent forms and have one week to think about their participation to the study. They can ask questions to the investigator (by email, phone or in consultation).

Patients and their legal representative come back after the reflection period. The investigator makes sure they have understood the information. They sign and date the informed consent/assent form simultaneously with the investigator.

Patients and their legal representative receive a copy of the signed informed consent form.

If patients or their legal representative refuse the study, they will receive the standard treatment as usual and they won't be included in the study.

Special requirements :

- Minors : Information would be given to the all parents, guardian or other mandated representative of the minor participant. The informed consent form would be signed by them. The minor should be involved in the process of informed consent, taking into account the age, the maturity degree (capacity of understanding) and the medical care, when selected to participate. The deliberate objection of a minor to take part to the experiment should always be respected even if the parents/representatives gave their consent.

Remarks : The participant's legal representative is the person designated by a written mandate dated and signed by both parties to represent the rights and defend the interests of the participant. If there is no legally designated person, the legal representative would be, in order,

the cohabitant (spouse, legal or effective), the adult child, the father or mother, the adult brother or sister.

Participants eligibility

For adolescents with mental disorders from AREA+ units
Inclusion criteria are: (a) aged between 12 and 19 years, (b) being admitted to the units of the psychiatric hospital of AREA+, (c) being able to stay focused on a task for 30 minutes based on the medical interview at the intake (d) having a proficient knowledge of French to allow them to fill out questionnaires and understand instructions, (e) living in Brussels, (f) inactive status, defined as not meeting the PA guidelines from World Health Organisation following the corresponding age (i.e. at least 420 min of Moderate and Vigorous Physical Activity (MVPA) /week for those aged 12-17 and at least 150 min of MVPA /week for those aged 18 and 19 years

Exclusion criteria are (a) planned hospitalisation during the intervention (b) physical disability preventing administration of standardised tests (e.g., inability to sit on a bicycle); (c) intellectual disability (defined as an intelligence quotient < 70); and (d) participants with serious medical problems or medical precautions prohibiting higher-intensity exercise (e.g., severe metabolic disorders, oncologic disease, unstable diabetes) according to the recommendations of the American College of Sports Medicine which are implemented within the clinical setting ; for the randomized controlled trial only. A general practitioner will cover the medical conditions and precautions for each participant at their hospitalisation in AREA+.

Withdrawal
Adolescents are free to withdraw from participation in the study at any time. The participant's request to withdraw from the study will always be respected and reasons to withdraw are not obliged to be mentioned, but will be requested to be able to report this information in the study. A subject must be discontinued from the study if the legal representative withdraws consent. We will use data collected up until the time of withdrawal.

An investigator may withdraw a subject from the study for the following reasons:

- Pregnancy being an exclusion criterion for the RCT, if a participant learns of her pregnancy during the intervention, under medical authorisation, she will be able to continue with the intervention but will be included in the drop-out list as her data will not be taken into account in the post-intervention or follow-up phases;
- Significant study non-compliance such refusing to complete the tests, repeated threatens with no changes after discussion with the PI on the collective functioning or integrity of the group and staff during the experiments;
- If any clinical adverse event (AE), laboratory abnormality, or other medical condition or situation occurs such that continued participation in the study would not be in the best interest of the subject;
- If the subject meets an exclusion criterion (either newly developed or not previously recognized) that precludes further study participation;
- Other : new physical injuries during the intervention period for the exercise group that prevent physical activity, new hospitalization during the intervention

In all cases, the reason why subjects are withdrawn must be recorded in detail in the electronic Case Report Form (eCRF) and in the subject's medical records. The gathered subject data should be taken into account in the analysis of the study data.

A subject will be considered lost to follow-up if he or she fails to return for the participation of creation process scheduled visits and for carrying out the co-created intervention and/or evaluation process and/or is unable to be contacted by the study site staff.
The following actions must be taken if a subject fails to return for a required study visit:

- The site will attempt to contact the subject and reschedule the missed visit within one week and counsel the subject on the importance of maintaining the assigned visit schedule and ascertain if the subject wishes to and/or should continue in the study;
- Before a subject is deemed lost to follow-up, the investigator or designee will make every effort to regain contact with the subject (i.e. three telephone calls and a certified letter to the subject's last known mailing address or local equivalent methods). These contact attempts should be documented in the subject's medical record or study file;
- Should the subject continue to be unreachable, he or she will be considered to have withdrawn from the study with a primary reason of lost to follow-up.

Patient follow-up after study participation

As all experimentations are added to usual care. As they will be in the current care, participants will continue to have a medical follow-up after study participation. If they are looking for any supplement support, we will suggest them potential contacts in the existing services in AREA+.

Start of the study

The study will start after ethical approval has been obtained. The study is considered started upon the first act of recruitment of a potential subject. For this study this is considered as date of the first screening in the patient's electronic file to assess whether the eligibility criteria have been met.

9. STUDY INTERVENTIONS AND PROCEDURES

Randomized controlled trial

Eligibility

Potential participants will be assessed for eligibility during the last week of hospitalisation or at the entrance in outpatient setting of AREA+. As described above the recruitment of participants will be done by the clinical and research assistant from units (residential units, day-to-day units and outpatient setting (=Consult-ado)) in AREA+ setting. Participants will be recruited by waves following the end of their hospitalisation and depending on meeting the inclusion criteria described above. All participants will provide their written informed assent and the written informed consent will be obtained from the parents/ legal representatives for minors.

Randomization

If they meet the inclusion criteria, we will proceed to the randomisation by minimisation based on age, sex, level of depression symptoms and physical activity level. Based on these parameters, we will allocate the participants randomly to the co-created exercise intervention or control group. This process will be performed after the pre-tests and before starting of intervention. More explanation is available below.

Baseline assessments

Baseline measurements will be collected during the first weeks following discharge or their start of outpatient care in AREA+.

Post-intervention assessments

Post-intervention measures will be done during the week after the intervention and follow-up assessments three months after the co-created intervention. Each evaluation time (same for the three times) consists of visits in Epsylon-AREA+ or with online mode.

Co-created intervention: BRIDGES

The intervention offers a comprehensive biopsychosocial approach to support adolescents with depression in integrating physical activity into their daily lives and has been designed followed the direct inputs of adolescent who emphasized on a true sense of freedom to choose their personalised mode of delivery. The BRIDGES intervention entails 18 sessions led by a physiotherapist during a 12-week period, illustrated in Figure 5. The first sessions aim to encourage open discussion, and create a supportive environment for learning and exchange between the adolescent and the physiotherapist counsellor. At the start, session frequency and delivery mode (online or face-to-face) will be also determined according to the adolescent's preferences over the three-month period of the intervention, allowing up to two remote sessions for one face-to-face session under the Belgian physiotherapy framework. The program combines individualized counselling with a structured transition to community-based physical activity opportunities. Counselling sessions, led by trained physiotherapists, will be grounded in evidence-based behaviour change frameworks, with a particular emphasis on Motivational Interviewing (MI) (67) and Self-Determination Theory (SDT) (68). These approaches are especially well-suited to working with adolescents experiencing depression, as they focus on enhancing internal motivation, autonomy, and personal agency. MI techniques will be used to engage participants in a collaborative, non-judgmental dialogue that supports their autonomy and helps resolve ambivalence about physical activity. Through reflective listening and open-ended questions, physiotherapists will help adolescents explore their values, goals, and motivations, building a sense of ownership over the change process. Informed by SDT, the counselling will also aim to foster three key psychological needs: autonomy (feeling in control of one's choices), competence (feeling capable and effective), and relatedness (feeling connected and supported). Sessions will be tailored to each adolescent's readiness, preferences, and personal circumstances, exploring individual barriers and strengths.

Relevant and inclusive opportunities from the AREA+ network, such as local sports clubs and initiatives, will be introduced in a way that aligns with the adolescent's interests, own choices and intrinsic motivation. To facilitate the transition to physical activity community, a network of inclusive clubs, associations, and initiatives will be developed based on a comprehensive mapping exercise focused around the AREA+ region. All participating clubs and associations will be carefully selected and provided with targeted training, under the request of adolescent in the co-creation, to ensure they can effectively support adolescents with depression. The training will focus on psychology and trauma-informed physical activity practice in order to offer a more inclusive and accessible participation. This training is the presented in this document as "training implementation".

The RCT protocol will be submitted to clinicalTrial.gov and a peer-reviewed journal when the acceptance of ethic committee will be delivered.

***Figure 5**: illustration of BRIDGES (Brussels Resilience and Inclusion for Depression – Guidance, Exercise and Support) intervention, co-created with inpatient adolescents*

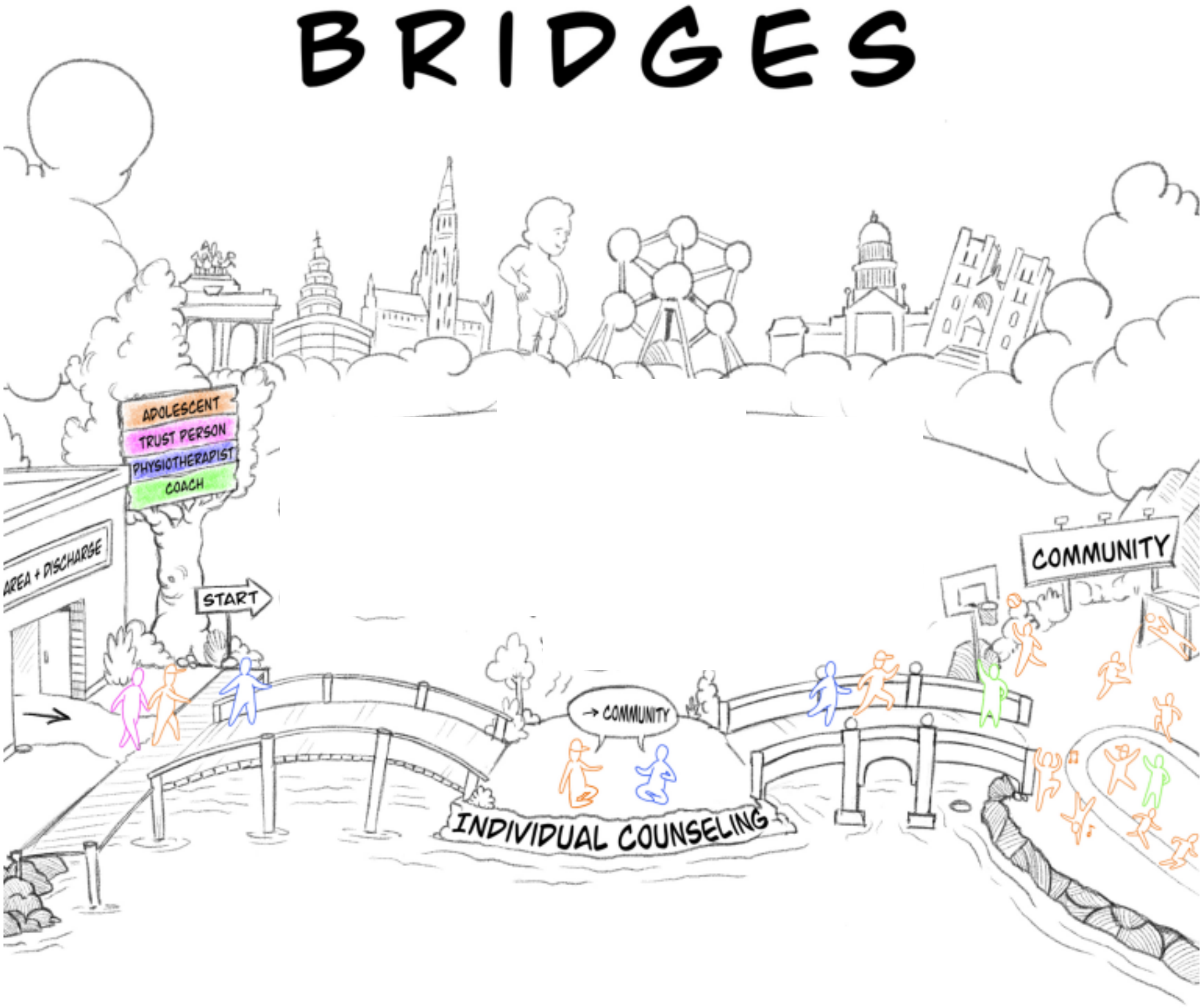

Control condition

The participants in the waitlist control group will follow the cares as usual after hospitalisation, being mainly psychological, social and medical follow-up. They still will be able to follow the intervention after 6 months (intervention + follow-up periods) and outside the study context.

Method of assigning participant to interventions groups

For the randomized controlled trial:

As the adolescents will be recruited in small waves following discharge, we must avoid the risk of obtaining unmatched groups at the end of the RCT and so, we will use randomisation and minimisation. Minimization is a randomization method that ensures balance of important prognostic factors between treatment groups without the disadvantages of stratification. It's considered as a dynamic and adaptive randomization technique since the randomization list is not produced before the trial

starts but during participant recruitment. Indeed, future participant allocation depends on previous assignments. So, we will match adolescents with the main criteria such as age, sex, level of depression symptoms and physical activity-levels, we will assign the participants randomly between both groups based on this method.

Protocol Deviations

Sponsor and all investigators agree to take any reasonable actions to correct protocol deviations/violations. All deviations must be documented on a protocol deviation log by the study team that is kept available at any time for audit/inspection purposes. Under emergency circumstances, deviations from the protocol to protect the rights, safety or well-being of human subjects may proceed without prior approval of the sponsor and the EC.

Data Quality Assurance

All study data will be handled in accordance with the law on General Data Protection Regulation (GDPR) and institutional rules [Belgian law dated on 20 July 2018 and 22 Aug. 2002]. The collection and processing of personal data from subjects enrolled in this study will be limited to those data that are necessary to fulfil the objectives of the study. These data must be collected and processed with adequate precautions to ensure confidentiality and compliance with applicable data privacy protection laws and regulations.

Appropriate technical and organizational measures to protect the personal data against unauthorized disclosures or access, accidental or unlawful destruction, or accidental loss or alteration must be put in place. Sponsor and site personnel whose responsibilities require access to personal data agree to keep the identity of subjects confidential.

The informed consent obtained from the subject includes explicit consent for the processing of personal data and for the investigator/institution to allow direct access to his or her original medical records (source data/documents) for study-related monitoring, audit, Ethics Committee review and regulatory inspection. This consent also addresses the transfer of the data to other entities, if applicable.

Privacy and confidentiality of data generated in the future on stored samples will be protected by the same standards applicable to all other clinical data. The investigator will ensure that the confidentiality of subjects' data will be preserved. On CRFs or any other documents, the subjects will not be identified by their names, but by their study number. Documents that identify the names of participants against their study number will be maintained by the investigator in strict confidence.

Monitors, auditors and other authorized agents will be granted direct access to study subject’s original medical records for verification of clinical study procedures and/or data, without violating the confidentiality of the subjects, to the extent permitted by the law and regulations. In any presentations of the results of this study at meetings or in publications, the subjects’ identity will remain confidential.

Statistical Analysis

Sample size and planned data analysis for the RCT are presented here.

Randomised controlled trial

Power analysis

As it's a pilot RCT with hybrid effectiveness-implementation design (type 2) (7), the expected number of participants is 30 per group for hybrid design (69). This pilot study will provide the effect size needed for a power analysis of a future RCT concerning quantitative data on effectiveness. For the qualitative data under the PRISM, we expect to recruit a purposive subsample of approximately 12 individuals to achieve data saturation for each interviewed stakeholder following implementation data collection under PRISM and RE-AIM (70). Recruitment will continue until data saturation is reached. In fact, as this is a pilot study conducted within a single mental health care setting, AREA+, the number of physiotherapists and clinicians/managers is expected to be limited and may not reach 12 participants per group.

Intervention analysis

Data analysis of quantitative measures

We will use a linear mixed model with F-test for differences between the different time of evaluation to contrast the outcomes of the post-care as usual regarding the outcomes for psychological, psycho-social and physical fitness. A statistician, hired for the analyses of this project, blind to the group allocation and to the identity of the patients, will carry out the statistical procedures using IBM SPSS Statistics 27.0 statistical software. Effects are reported with their 95% confidence interval (95%CI). A p-value lower than 0.05 is considered as significant. During the follow-up evaluation, we will analyse odds ratio of any re-hospitalisation and their pathological threshold score in psychological assessments revealing also a relapse. An odds ratio is a measure of association between an exposure and an outcome. The odds ratio represents the odds that an outcome (re-hospitalisation) will occur given a particular exposure (post-care as usual and co-created intervention), compared to the odds of the outcome occurring in the absence of that exposure (post-care as usual only).

Data analysis of qualitative measures

Data will be analyzed using the Qualitative Analysis Guide of Leuven (QUAGOL) (71), a method inspired by the Grounded Theory Approach (72). The QUAGOL method facilitates a comprehensive examination of qualitative interview data. The initial phase, termed "paper and pencil work," precedes the coding process and serves as a preparatory stage. The subsequent phase involves coding using specialized software. Transcripts will be entered and analysed into Nvivo for qualitative data analysis by multiple researchers independently. Data will be presented according to the Consolidated Criteria for Reporting Qualitative Research (COREQ) checklist (73).

Protocol Amendments

If amendments to the protocol (modifying sense or objectives or modifying the undergone constraints or the risks incurred by the subjects) turn out to be necessary, they will be subjected at first opinion of the promoter of the study. After agreement by the promoter, these amendments will then be submitted to the opinion of the Ethic Committee having examined the initial protocol.

As expected, an amendment is now sent with the current version to the Ethic Committee to describe and adapt the protocol from the creation of the intervention that will be evaluated under RCT at the end of the co-creation process.

Data Management Responsibilities

Data handling and record keeping

Subjects who are included in the study will be assigned a unique study number. On all documents submitted to the sponsor, patients will only be identified by their study number. The subject identification list will be safeguarded by the site. The name and any other directly identifying details will not be included in the study database.

An electronic case report form (eCRF) will be used in REDCap software. The eCRF will be completed for subjects who have signed the informed consent. This eCRF will include specific pages for inclusion and exclusion criteria, and for reporting each visit. Other specific pages will be dedicated to concomitant treatments and AEs (non-serious and serious). The investigator will review, approve and validate each completed eCRF; the investigator's signature (validation) serving as attestation of the investigator's responsibility for ensuring that all data entered on the eCRF are complete, accurate and authentic.

All data will be processed according to the principles that the new European General Data Protection Regulation (GDPR) imposes, which is in force since 25 May 2018.

1. Who will responsible for the processing of personal data?
   Arnaud Philippot

2. Who is Data Protection Officer for the processing?
The institutional DPO could be reached by this email address : privacy@uclouvain.be

3. The purpose of the processing:
   - Scientific research

4. The legal basis of the processing:
   - Consent, but this can be withdrawn

4. Who are potential recipients of the personal data?
   - All researchers involved in this clinical study or in research projects that use materials original from this clinical study. Staff involved in monitoring and ethical evaluation and people from competent authorities. Subcontracted parties that perform analysis on study-related data or materials.

5. It is possible that the personal data will be viewed by people who are in countries that do not use the same standards as the EU in terms of legal protection of data. In that case, we guarantee that the conditions of European and Belgian legislation on the protection of personal data will be respected.

6. The storage period:
   - Study-related documents will be stored for at least 20 years, data included in the medical file for 30 years.

Case Report Form

An electronic data capture (EDC) system, i.e. REDCap, will be used for data collection. Data reported on each eCRF should be consistent with the source data. If information is not known, this must be clearly indicated on the eCRF. All missing and ambiguous data will be clarified.
The eCRFs will be developed, based on the protocol. The final eCRF design will be approved by the Coordinating Investigator.

All data entries and corrections will only be performed by study site staff, authorized by the investigator. Data will be checked by trained personnel (monitor) and any errors or inconsistencies will be clarified. The investigator must verify that all data entries in the eCRF are accurate and correct.

REDCap is provided and maintained by Vanderbilt University; a license for use was granted to the UCLouvain. REDCap is a web-based system.

Data storage
The data is accessed through a web browser directly on the secure REDCap server. The server is hosted within the UCLouvain campus and meets high level security and back-up requirements.

Access to data
Direct access will be granted to authorised representatives from the Sponsor, host institution and the regulatory authorities to permit study-related monitoring, audits and inspections.
Login in REDCap is password controlled. Each user will receive a personal login name and password and will have a specific role which has predefined restrictions on what is allowed in REDCap. Any activity in the software is traced and transparent via the audit trail and log files.

Data breach
Research data management will follow the annexed “Research Collaboration Agreement”, and signed by UCLouvain, AREA+ Epsylon ASBL and KULeuven. All research data will be treated confidentially and will be pseudo-anonymized. All data will be stored on a secure server through a RedCap database.

- This project will have 6 types of data: (a) Informed assent/consent forms (digital; REDCap); (b) Demographic data (age, sex, health inequalities, declared gender, date of birth; digital; REDCap); (c) Surveys/questionnaires (digital: REDCap); (e) Clinical data (diagnosis, medication use; fitness levels; digital; REDCap); (f) Interviews: audio-recorded, transcribed, analysed (NVivo 12 software).
- UCLouvain and KU Leuven will be responsible for data management. De-identified hard copies will be stored in a secured locker in a locked room at UCLouvain that can only be accessed by authorised personnel. Electronic data (all coded and containing no personal information) will be stored on secured servers that are double password and site firewall protected. The REDCap database management system from UCLouvain will be used to store all electronic case report forms of the study participants. Participants’ contact information will be stored in an independent secured database. All data will be kept for at least 20 years after publication.
- Data gathered in this research is sensitive data of the participants. Therefore, measures will be taken to de-identify the data. Only researchers involved in the project will be able to trace back the data to the participant, for instance, using personal identification codes for the participants. Only the project supervisor will have full access to the patient data. All other investigators will have limited REDCap access, allowing them to only consult patient information that is relevant for their role in this research project.

The Sponsor or designee must report to ethics committee, serious data breaches : transgressions against the study protocol or the regulation that are likely to significantly affect the safety and rights of a subject or the reliability and robustness of the data generated in the study.

10. Finance and Insurance

The experimentation is covered under the Belgian Law of May 7, 2004 by a no-fault insurance (type of coverage: liability insurance).

For the 60 outpatients taking part in the randomised controlled trial, the cost of transport will be reimbursed and attendance at each assessment session will be indemnified with 15 euros per evaluation time.

The sessions in the RCT doesn't include the participation of adolescents among the local clubs.

Policy holder:
UCLouvain
Place de l'Université 1
1348 Louvain-la-Neuve

Issuer of the certificate of insurance:
MS Amlin Insurance SE
Boulevard du Roi Albert II, 37
1030 Brussels
N° de police : LXX111372

11. End of study

For an individual subject

The subject has completed the study if he or she has completed all of study procedures, including the last visit or the last scheduled procedure, as described in this protocol (see section "Study Specific Procedures".

For the whole study

Overall, the end of the study is reached when the last study procedure for the last subject has occurred: last subject, last visit (LSLV).
As soon as the whole study has ended (cfr the definition above), the coordinating Investigator shall notify the sponsor, so that the Ethics Committee can be informed in a timely manner according to the regulatory requirements (within 90 days after the end of the study, or if the study had to be terminated early, this period must be reduced to 15 days and the reasons should clearly explained).

12. Dissemination of Results and Publication Policy

This study will be registered on ClinicalTrials.gov (https://clinicalTrials.gov/) and will be available to the public when the co-creation process will be performed.

13. Archiving

Essential clinical study documents are kept at least 20 years after the study termination according to the Belgian legislation: RD 18 May 2006 art.24.

Source documentation are kept for 30 years, according to the Belgian legislation (Art 35 Belgian Law of 22 April 2019).

14. Study Report

The entire project will last three years and at the end, a final report, written by Arnaud Philippot, will be sent to Saint-Luc ethic committee and the local ethic committee of Epsylon.

15. Literature References

1. Patel AV, Friedenreich CM, Moore SC, Hayes SC, Silver JK, Campbell KL, et al. American College of Sports Medicine Roundtable Report on Physical Activity, Sedentary Behavior, and Cancer Prevention and Control. Med Sci Sports Exerc. 2019;51(11):2391–402.
2. Caspersen CJ, Powell KE, Christenson GM. Physical activity, exercise, and physical fitness: definitions and distinctions for health-related research. Public health reports. 1985;100(2):126.
3. Roser T, Samson A, Humphreys P, Cruz-Valdivieso E. Co-creation: new pathways to value: an overview. Promise & LSE Enterprise. 2009.
4. Greenhalgh T, Jackson C, Shaw S, Janamian T. Achieving research impact through co-creation in community-based health services: literature review and case study. The Milbank Quarterly. 2016;94(2):392–429.
5. Proctor E, Silmere H, Raghavan R, Hovmand P, Aarons G, Bunger A, et al. Outcomes for implementation research: conceptual distinctions, measurement challenges, and research agenda. Administration and policy in mental health and mental health services research. 2011;38(2):65–76.
6. Lewis CC, Fischer S, Weiner BJ, Stanick C, Kim M, Martinez RG. Outcomes for implementation science: an enhanced systematic review of instruments using evidence-based rating criteria. Implementation Science. 2015;10(1):155.
7. Landes SJ, McBain SA, Curran GM. Reprint of: an introduction to effectiveness-implementation hybrid designs. Psychiatry research. 2020;283:112630.
8. Golightly YM, Allen KD, Ambrose KR, Stiller JL, Evenson KR, Voisin C, et al. Physical Activity as a Vital Sign: A Systematic Review. Prev Chronic Dis. 2017;14:E123.
9. Roempler J, Petzold MB, Bendau A, Plag J, Ströhle A. Tracking changes in physical activity during inpatient treatment in a psychiatric clinic in Germany by asking two simple questions. European Archives of Psychiatry and Clinical Neuroscience. 2023;273(4):983–94.
10. Vancampfort D, Mugisha J, Van Damme T. The Physical Activity Vital Sign and associations with cardiometabolic risks in people with bipolar disorder in a low-income country. Journal of Affective Disorders. 2023;339:763–6.
11. Nelson VR, Masocol RV, Asif IM. Associations between the physical activity vital sign and cardiometabolic risk factors in high-risk youth and adolescents. Sports Health. 2020;12(1):23–8.
12. Nandakumar AL, Vande Voort JL, Nakonezny PA, Orth SS, Romanowicz M, Sonmez AI, et al. Psychometric Properties of the Patient Health Questionnaire-9 Modified for Major Depressive Disorder in Adolescents. J Child Adolesc Psychopharmacol. 2019;29(1):34–40.
13. Fonseca-Pedrero E, Díez-Gómez A, Pérez-Albéniz A, Al-Halabí S, Lucas-Molina B, Debbané M. Youth screening depression: Validation of the Patient Health Questionnaire-9 (PHQ-9) in a representative sample of adolescents. Psychiatry Res. 2023;328:115486.
14. Liu FF, Adrian MC. Is Treatment Working? Detecting Real Change in the Treatment of Child and Adolescent Depression. J Am Acad Child Adolesc Psychiatry. 2019;58(12):1157–64.
15. Feldstein AC, Glasgow RE. A practical, robust implementation and sustainability model (PRISM) for integrating research findings into practice. The joint commission journal on quality and patient safety. 2008;34(4):228–43.
16. Stoutenberg M, Galaviz KI, Lobelo F, Joy E, Heath GW, Hutber A, et al. A pragmatic application of the RE-AIM framework for evaluating the implementation of physical activity as a standard of care in health systems. Preventing chronic disease. 2018;15:E54.

17. Teasdale SB, Machaczek K, Marx W, Eaton M, Chapman J, Milton A, et al. Implementing lifestyle interventions in mental healthcare: the third report from The Lancet Psychiatry Physical Health Commission. The Lancet Psychiatry. 2025.
18. Vancampfort D, Firth J, Stubbs B, Schuch F, Rosenbaum S, Hallgren M, et al. The efficacy, mechanisms and implementation of physical activity as an adjunctive treatment in mental disorders: a meta-review of outcomes, neurobiology and key determinants. World Psychiatry. 2025;24(2):227–39.
19. Fiorillo A, Firth J, Misiak B, Rojnic Kuzman M, Samochowiec J, Sampogna G, et al. From lifestyle psychiatry to whole person health: evidence, dissemination and scalable approaches. European Psychiatry. 2025:1–10.
20. Li X, Jin W, Han L, Chen X, Li L. Comparison and application of depression screening tools for adolescents: scale selection and clinical practice. Child and Adolescent Psychiatry and Mental Health. 2025;19(1):53.
21. Beck AT, Steer RA, Brown G. Beck depression inventory–II. Psychological assessment. 1996.
22. Gartland D, Bond L, Olsson CA, Buzwell S, Sawyer SM. Development of a multi-dimensional measure of resilience in adolescents: the Adolescent Resilience Questionnaire. BMC Medical Research Methodology. 2011;11(1):134.
23. Bruwer B, Emsley R, Kidd M, Lochner C, Seedat S. Psychometric properties of the Multidimensional Scale of Perceived Social Support in youth. Compr Psychiatry. 2008;49(2):195–201.
24. Cecil H, Stanley MA, Carrion PG, Swann A. Psychometric properties of the MSPSS and NOS in psychiatric outpatients. J Clin Psychol. 1995;51(5):593–602.
25. Laksmita OD, Chung M-H, Liao Y-M, Chang P-C. Multidimensional Scale of Perceived Social Support in Indonesian adolescent disaster survivors: A psychometric evaluation. PloS one. 2020;15(3):e0229958.
26. Resnick B, Jenkins LS. Testing the reliability and validity of the self-efficacy for exercise scale. Nursing research. 2000;49(3):154–9.
27. Yao L, Zhou K, Zhou Y, Kueh YC, Liu H, Liu Z, et al. Psychometric properties of the Chinese version of the Exercise Self-Efficacy Scale for the Transtheoretical Model: a confirmatory analysis among Chinese children and adolescents. BMC Public Health. 2024;24(1):151.
28. Donato ANA, Waclawovsky AJ, Tonello L, Firth J, Smith L, Stubbs B, et al. Association between cardiorespiratory fitness and depressive symptoms in children and adolescents: A systematic review and meta-analysis. Journal of affective disorders. 2021;282:1234–40.
29. Raghuveer G, Hartz J, Lubans DR, Takken T, Wiltz JL, Mietus-Snyder M, et al. Cardiorespiratory Fitness in Youth: An Important Marker of Health: A Scientific Statement From the American Heart Association. Circulation. 2020;142(7):e101–e18.
30. Correll CU, Solmi M, Veronese N, Bortolato B, Rosson S, Santonastaso P, et al. Prevalence, incidence and mortality from cardiovascular disease in patients with pooled and specific severe mental illness: a large-scale meta-analysis of 3,211,768 patients and 113,383,368 controls. World Psychiatry. 2017;16(2):163–80.
31. Firth J, Siddiqi N, Koyanagi A, Siskind D, Rosenbaum S, Galletly C, et al. The Lancet Psychiatry Commission: a blueprint for protecting physical health in people with mental illness. The Lancet Psychiatry. 2019;6(8):675–712.
32. Laukkanen JA, Isiozor NM, Kunutsor SK. Objectively Assessed Cardiorespiratory Fitness and All-Cause Mortality Risk: An Updated Meta-analysis of 37 Cohort Studies Involving 2,258,029 Participants. Mayo Clin Proc. 2022;97(6):1054–73.
33. Sciensano. Sixième enquête de santé COVID-19. Bruxelles, Belgique ; Numéro de dépôt :D/2021/14.440.30. Disponible en ligne : https://doi.org/10.25608/j877-kf56.
34. Shorey S, Ng ED, Wong CH. Global prevalence of depression and elevated depressive symptoms among adolescents: A systematic review and meta-analysis. British Journal of Clinical Psychology. 2022;61(2):287–305.

35. Bockting CL, Hollon SD, Jarrett RB, Kuyken W, Dobson K. A lifetime approach to major depressive disorder: the contributions of psychological interventions in preventing relapse and recurrence. Clinical psychology review. 2015;41:16–26.
36. Curry J, Silva S, Rohde P, Ginsburg G, Kratochvil C, Simons A, et al. Recovery and Recurrence Following Treatment for Adolescent Major Depression. Archives of General Psychiatry. 2011;68(3):263–9.
37. Korczak DJ, Ofner M, LeBlanc J, Wong S, Feldman M, Parkin PC. Major Depressive Disorder Among Preadolescent Canadian Children: Rare Disorder or Rarely Detected? Acad Pediatr. 2017;17(2):191–7.
38. Vancampfort D, Van Damme T, Stubbs B, Smith L, Firth J, Hallgren M, et al. Sedentary behavior and anxiety-induced sleep disturbance among 181,093 adolescents from 67 countries: a global perspective. Sleep Med. 2019;58:19–26.
39. Philippot A, Dubois V, Lambrechts K, Grogna D, Robert A, Jonckheer U, et al. Impact of physical exercise on depression and anxiety in adolescent inpatients: A randomized controlled trial. Journal of affective disorders. 2022.
40. Recchia F, Bernal JD, Fong DY, Wong SH, Chung P-K, Chan DK, et al. Physical Activity Interventions to Alleviate Depressive Symptoms in Children and Adolescents: A Systematic Review and Meta-analysis. JAMA pediatrics. 2023.
41. Carter T, Pascoe M, Bastounis A, Morres ID, Callaghan P, Parker AG. The effect of physical activity on anxiety in children and young people: A systematic review and meta-analysis. Journal of Affective Disorders. 2021;285:10–21.
42. Michie S, Atkins L, West R. The behaviour change wheel. A guide to designing interventions 1st ed Great Britain: Silverback Publishing. 2014;1003:1010.
43. Deci EL, Ryan RM. Self-determination theory. Handbook of theories of social psychology. 2012;1(20):416–36.
44. Marent P-J, Vangilbergen A, Chastin S, Cardon G, van Uffelen JGZ, Beeckman M. Conceptualization of a cognitively enriched walking program for older adults: a co-design study with experts and end users. BMC Geriatrics. 2022;22(1):167.
45. Maenhout L, Verloigne M, Cairns D, Cardon G, Crombez G, Melville C, et al. Co-creating an intervention to promote physical activity in adolescents with intellectual disabilities: lessons learned within the Move it, Move ID!-project. Res Involv Engagem. 2023;9(1):10.
46. Sofro ZM, Wibowo RA, Wasityastuti W, Kusumadewi AF, Utomo PS, Ekawati FM, et al. Physical activity virtual intervention for improving mental health among university students during the COVID-19 pandemic: A Co-creation process and evaluation using the Behavior Change Wheel. Heliyon. 2023;9(8):e18915.
47. Nasstasia Y, Baker AL, Lewin TJ, Halpin SA, Hides L, Kelly BJ, et al. Differential treatment effects of an integrated motivational interviewing and exercise intervention on depressive symptom profiles and associated factors: A randomised controlled cross-over trial among youth with major depression. Journal of affective disorders. 2019;259:413–23.
48. O’Halloran PD, Blackstock F, Shields N, Holland A, Iles R, Kingsley M, et al. Motivational interviewing to increase physical activity in people with chronic health conditions: a systematic review and meta-analysis. Clinical rehabilitation. 2014;28(12):1159–71.
49. Hardcastle SJ, Taylor AH, Bailey MP, Harley RA, Hagger MS. Effectiveness of a motivational interviewing intervention on weight loss, physical activity and cardiovascular disease risk factors: a randomised controlled trial with a 12-month post-intervention follow-up. International journal of behavioral nutrition and physical activity. 2013;10(1):1–16.
50. Czosnek L, Rosenbaum S, Rankin NM, Zopf EM, Cormie P, Herbert B, et al. Implementation of physical activity interventions in a community-based youth mental healthcare service: A case study of context, strategies, and outcomes. Early Interv Psychiatry. 2023;17(2):212–22.
51. Lancet T. Social prescribing: bringing community back to health? 2025. p. 103.

52. Björkman F, Eggers A, Stenman A, Bohman T, Ekblom B, Ekblom Ö. Sex and maturity status affected the validity of a submaximal cycle test in adolescents. Acta Paediatrica. 2018;107(1):126–33.
53. Kandola A, Lewis G, Osborn DPJ, Stubbs B, Hayes JF. Depressive symptoms and objectively measured physical activity and sedentary behaviour throughout adolescence: a prospective cohort study. The lancet Psychiatry. 2020;7(3):262–71.
54. Das JK, Salam RA, Lassi ZS, Khan MN, Mahmood W, Patel V, et al. Interventions for Adolescent Mental Health: An Overview of Systematic Reviews. Journal of Adolescent Health. 2016;59(4, Supplement):S49–S60.
55. Maes S. «On connaît une vague de décompensation psychique inédite chez les 13-25 ans» (Dr Sophie Maes). Belga 2022.
56. Luxton R, Kyriakopoulos M. Depression in children and young people: identification and management NICE guidelines. Archives of Disease in Childhood-Education and Practice. 2022;107(1):36–8.
57. Heissel A, Heinen D, Brokmeier LL, Skarabis N, Kangas M, Vancampfort D, et al. Exercise as medicine for depressive symptoms? A systematic review and meta-analysis with meta-regression. British Journal of Sports Medicine. 2023.
58. Schuch FB, Stubbs B, Meyer J, Heissel A, Zech P, Vancampfort D, et al. Physical activity protects from incident anxiety: A meta-analysis of prospective cohort studies. Depression and anxiety. 2019;36(9):846–58.
59. Martland R, Korman N, Firth J, Stubbs B. The efficacy of exercise interventions for all types of inpatients across mental health settings: A systematic review and meta-analysis of 47 studies. J Sports Sci. 2023:1–40.
60. Salvo D, Garcia L, Reis RS, Stankov I, Goel R, Schipperijn J, et al. Physical activity promotion and the United Nations sustainable development goals: building synergies to maximize impact. Journal of physical activity and health. 2021;18(10):1163–80.
61. de Camargo EM, Chen S, López-Bueno R, Mesas AE, Bizzozero-Peroni B, Martín-Calvo N, et al. Are perceived barriers to physical activity related to depression, anxiety and stress among adolescents? The EHDLA study. BMJ Open Sport & Exercise Medicine. 2024;10(3).
62. Sunesson E, Haglund E, Bremander A, Jarbin H, Larsson I. Adolescents’ experiences of facilitators for and barriers to maintaining exercise 12 months after a group-based intervention for depression. International journal of environmental research and public health. 2021;18(10):5427.
63. Timlin U, Hakko H, Heino R, Kyngäs H. Factors that Affect Adolescent Adherence to Mental Health and Psychiatric Treatment: a Systematic Integrative Review of the Literature. Scandinavian Journal of Child and Adolescent Psychiatry and Psychology. 2015;3(2):99–107.
64. Stubbs B, Vancampfort D, Rosenbaum S, Ward PB, Richards J, Soundy A, et al. Dropout from exercise randomized controlled trials among people with depression: A meta-analysis and meta regression. J Affect Disord. 2016;190:457–66.
65. Vancampfort D, Stubbs B, Venigalla SK, Probst M. Adopting and maintaining physical activity behaviours in people with severe mental illness: The importance of autonomous motivation. Preventive medicine. 2015;81:216–20.
66. Stubbs B, Vancampfort D, Hallgren M, Firth J, Veronese N, Solmi M, et al. EPA guidance on physical activity as a treatment for severe mental illness: a meta-review of the evidence and Position Statement from the European Psychiatric Association (EPA), supported by the International Organization of Physical Therapists in Mental Health (IOPTMH). European Psychiatry. 2018;54:124–44.
67. Miller WR, Rollnick S. Motivational interviewing: helping people change and grow. (No Title). 2023.
68. Deci EL, Ryan RM. Self-determination theory. 2012.
69. Moore GF, Audrey S, Barker M, Bond L, Bonell C, Hardeman W, et al. Process evaluation of complex interventions: Medical Research Council guidance. BMJ : British Medical Journal. 2015;350:h1258.

70. Guest G, Bunce A, Johnson L. How Many Interviews Are Enough?:An Experiment with Data Saturation and Variability. Field Methods. 2006;18(1):59–82.
71. De Casterlé BD, Gastmans C, Bryon E, Denier Y. QUAGOL: A guide for qualitative data analysis. International journal of nursing studies. 2012;49(3):360–71.
72. Charmaz K. Constructing grounded theory: A practical guide through qualitative analysis: sage; 2006.
73. Tong A, Sainsbury P, Craig J. Consolidated criteria for reporting qualitative research (COREQ): a 32-item checklist for interviews and focus groups. International journal for quality in health care. 2007;19(6):349–57.

16. Annexe

Research Collaboration Agreement
Formulaire de Rapport de Cas (FRC) - Événement Indésirable

Questionnaires for the amendment :
-PHQ-9M
-PAVS
-ARQ-M
-MSPSS
-SEE
